# Genetic analyses enabled by the fourth chromosome resource project reveal unexpected mutant phenotypes and suggest new disease models

**DOI:** 10.1093/g3journal/jkag077

**Published:** 2026-03-28

**Authors:** Bonnie M Weasner, Michael J Stinchfield, Brandon P Weasner, Saeko Takada, Michael B O’Connor, Justin P Kumar, Stuart J Newfeld

**Affiliations:** Department of Biology, Indiana University, Bloomington, IN 47405, United States; School of Life Sciences, Arizona State University, Tempe, AZ 85287-4501, United States; Department of Biology, Indiana University, Bloomington, IN 47405, United States; Department of Biochemistry, Molecular Biology, & Biophysics, University of Minnesota, Minneapolis, MN 55455, United States; Department of Genetics, Cell Biology & Development, University of Minnesota, Minneapolis, MN 55455, United States; Department of Biology, Indiana University, Bloomington, IN 47405, United States; School of Life Sciences, Arizona State University, Tempe, AZ 85287-4501, United States

**Keywords:** eyeless, legless, maverick, GRM1, YY1, Dros2026

## Abstract

To date, the Fourth Chromosome Resource Project (FCRP) has deposited over 850 stocks for the genetic analysis of its 79 protein-coding genes. Here, we employ those stocks to reveal unexpected phenotypes for multiple exemplar genes. Expression studies of the transcript and protein provide clues to an adult function for *maverick*, a gene that, despite prior efforts, has remained inscrutable. Loss-of-function studies reveal an adult brain phenotype for the well-studied signal transducer *legless* and the first phenotype for *datilographo*, a gene with no prior mutations. Marked clones with a new *eyeless* null allele in the larval brain elicited the first heterochronic phenotype in flies. Mutant clones of *myoglianin* encompassing the entire adult glial blood-brain barrier elicited overgrowth of the underlying optic lobes. Complete clones of one glial layer within the two-layer barrier were obtained for multiple genes and provide an opportunity to interrogate barrier crossing mechanisms for neurological therapeutics. In overexpression studies, rough eye phenotypes were generated by two human genes known to cause autosomal dominant neurological diseases. How YY1 and GRM1 haploinsufficiency leads to inherited cognitive impairment and spinocerebellar ataxia visible from birth is currently unknown. Fly eye phenotypes provide a tractable disease model for understanding their mechanism of action. Taken together, the ease with which mutant phenotypes were revealed suggests that a considerable amount of interesting biology remains to be uncovered on the fourth chromosome.

## Introduction

At the start of the Fourth Chromosome Resource Project (FCRP) fewer than 50% of the 79 protein-coding genes on the fourth chromosome had a mutation. The majority of genes were known simply by annotation of the genome sequence, and their potential biochemical functions were predicted by sequence similarity to previously studied proteins. Recently, we reported efforts to directly mutagenize every protein-coding gene on the fourth chromosome via CRISPR (Cas9-mediated homology-directed repair), with the mutated gene located on a chromosome containing a proximal FRT site (Flippase recognition target; Goldsmith et al. 2022; Stinchfield et al. 2024; Weasner et al. 2025). These mutations allow for analysis of organismal impacts of the mutation as well as single-cell impacts of the mutation when employing mitotic clones. At present, all of the protein-coding genes on the fourth chromosome have a mutation, though not quite all (96%) have one on the FRT chromosome. The other FCRP stock collections are at or just below this level of completeness.

The FCRP also adds value to these collections by conducting validation assays. Validation assays identified unexpected issues with a legacy UAS fly cDNA vector and our original Tub.GAL80 insertion that were addressed immediately. These issues and their solutions are noted below. Our remedy for the problem of fourth triploidy was recently published (Cook 2025). We demonstrate the utility of our stocks by analyzing selected fourth chromosome genes in detail, such as *apolipophorin* (Stinchfield et al. 2026). We analyze many stocks at the single-experiment level to suggest clues to previously unknown functions. For fourth chromosome genes where the FCRP has generated stocks containing a UAS fly cDNA and stocks with a UAS human ortholog cDNA, we conducted pairwise validation assays in larvae employing an eye-specific GAL4 driver. These overexpression studies often elicited phenotypes impacting a specific cell type. In two cases, human genes that are the cause of autosomal dominant neurological disorders and whose mechanism of action is unknown, generated distinct photoreceptor defects. A fly eye photoreceptor defect allows for the application of sophisticated genetic tools to uncover what went wrong, as a clue to the neurological function of each human gene.

We illustrate our validation studies with examples including: two highly conserved Transforming Growth Factor-β family members (*maverick* and *myoglianin*), three fly and human orthologous pairs (*pleiohomeotic*/YY1, *metabotropic Glutamate Receptor*/GRM1 and *onecut*/ONECUT1), two highly conserved Paired box proteins (*eyeless* and *twin of eyeless*), and three transcription factors (*datilographo*, *legless* and *zfh2*). We analyze these genes either in the third instar larvae to shed light on developmental roles or in the adult brain to better understand homeostasis. The full range of FCRP stocks is employed in these illustrations, demonstrating the ability to study genes/mutations on the fourth chromosome as if they were on any other chromosome.

## Materials and methods

### Genetic conversion of CRIMIC transgenes then GFP validation in brains

See Supplementary Table 1 for a glossary of current genomic technology terms. Genetic conversions were initially focused on MiMIC transgenes (Minos Mediated Integration Cassette; Nagarkar-Jaiswal et al. 2015). Then we focused on CRISPR-mediated integration cassette transgenes (CRIMIC; Lee et al. 2018). Both sets of converted transgenes were located in an intron within a gene's protein-coding region. Conversions were conducted with a DoubleHeader donor transgene via Recombination Mediated Cassette Exchange crossing schemes (RMCE; Li-Kroeger et al. 2018). Matching the DoubleHeader donor reading frame with the gene's open reading frame in these crosses resulted in the conversion of the CRIMIC transgene to either an in-frame eGFP tag (protein trap) or an in-frame T2A.GAL4 sequence (gene trap). Conversion occurs via Cre-loxP excision of the combined eGFP and T2A.GAL4 artificial exons from the DoubleHeader donor. This is followed by C31-dependent recombination of the eGFP and T2A.GAL4 artificial exon attB sites with intronic CRISPR attP sites (Bischof et al. 2007). The result is a swap of the eGFP and T2A.GAL4 artificial exons from the donor for the CRIMIC 3×P3-GFP transformation marker. The orientation of the inserted donor eGFP and T2A.GAL4 artificial exon will place either the eGFP or T2A.GAL4 sequence in frame with the endogenous protein, leading to their expression in the pattern of the endogenous promoter.

The eGFP artificial exon contains a splice donor and a splice acceptor flanking a version of the Green Fluorescent Protein (GFP) with amino acid substitutions that emit 35 times more fluorescence than native GFP (Yang et al. 1996). The T2A.GAL4 artificial exon has a splice donor and a splice acceptor flanking a 2A peptide from the insect virus *Thosea asigna* that causes ribosomal skipping. The skipping process terminates the translation of the endogenous protein and initiates the translation of the transcription factor GAL4 (Diao and White 2012; Diao et al. 2015). Thus, the T2A.GAL4 insertion creates both a loss-of-function mutation in the endogenous gene and drives translation of GAL4 in the expression pattern of the endogenous gene's mRNA.

Immunohistochemistry to detect expression of either the eGFP-tagged protein or expression of T2A.GAL4 driving UAS.nls-GFP (GFP with a nuclear localization signal attached to focus expression) was conducted as described (Stinchfield et al. 2024). For select CRIMICs, FCRP conversion to DoubleHeader T2A.GAL4 was verified for use in our labs, but the stocks were declined for curation by Bloomington. The reason is that CRIMIC transgenes have their own T2A.GAL4 artificial exon and the DoubleHeader converted T2A.GAL4 stocks were considered redundant. The value to us of a DoubleHeader converted T2A.GAL4 stock is that the green channel is available for experiments rather than being occupied by the 3×P3-GFP transformation marker in a CRIMIC. If you have a need for a fourth CRIMIC converted to DoubleHeader T2A.GAL4 to allow experiments in the green channel, then contact the FCRP for the crossing scheme. While RMCE may sound daunting, we have shared the crossing scheme with colleagues who reported successful conversion. Supplementary Fig. 1 contains images of eGFP converted stocks not available previously, with brief descriptions in the legend. A list of recently converted stocks with their Bloomington and Kyoto stock numbers is in Supplementary Table 2.

### 3′-HA tagged UAS fly and UAS human cDNA stocks validated in eye discs and adult eyes

Fly and human cDNAs were cloned into an attB-containing UAS vector. This vector adds a hemagglutinin (HA) tag to the 3′ end of cDNAs that had their endogenous stop codon removed (pGW-HA.attB; Bischof et al. 2013). Cloned cDNAs were co-injected with ϕC31 integrase that inserts the UAS cDNA-3′-HA sequence by recombination between the vector attB sites and genomic attP sites on chromosome 2 (VK37; BL9752) or on chromosome 3 (VK33; BL9750; Venken et al. 2006). These insertion sites are also the locations of UAS human cDNAs generated by the Gene Disruption Project (Yamamoto et al. 2014). The prefix BL indicates a Bloomington stock number that is also a Research Resource Identifier (RRID). Immunohistochemistry followed Stinchfield et al. (2024). All UAS cDNAs with a 3′-HA tag (endogenous stop codon removed) had their 3′-HA expression validated in larval eye discs. An update with new UAS fly and UAS human cDNA stocks is in Supplementary Table 3. New images for 3′-HA tagged UAS fly cDNA stocks are in Supplementary Fig. 2, and new images for 3′-HA tagged UAS human cDNA stocks are in Supplementary Fig. 3. A summary of 3′-HA expression data for UAS fly and UAS human cDNA stocks is in Supplementary Table 4.

For 3′-HA expression tests and identifying adult eye phenotypes, the following two GAL4 driver stocks were utilized: P{w[ + mC] = GAL4-ninaE.GMR}12 (BL1104; abbreviated GMR.GAL4) and *eyes absent* composite enhancer GAL4 (Weasner et al. 2016). Third instar eye-antennal imaginal discs were dissected and prepared as described (Spratford and Kumar 2014). Primary antibodies for UAS cDNA 3′-HA tag validation and overexpression phenotype analyses were: rat α-HA (Roche 3F10, 1:500), mouse α-Elav (DSHB 7E8A10, 1:100), mouse α-Eya (DSHB eya10H6, 1:5), mouse α-Cut (DSHB 2B10, 1:100), mouse α-LacZ (Promega Z3781, 1:250), and rabbit α-GFP (Abcam 6556, 1:1,000). Far Red Phalloidin (650 nm; ThermoFisher #A22287, 1:20) and Rhodamine Phalloidin (565 nm; ThermoFisher #R415, 1:100) were also employed. The following secondary antibodies were obtained from Jackson ImmunoResearch: Fluorescein Donkey α-Rat (712-095-153, 1:100), Cy3 Donkey α-Mouse (715-165-151, 1:100), and Cy3 Donkey α-Rat (712-165-153, 1:100). Fluorescent images of eye discs were taken with a Zeiss Axioplan II and processed with Zeiss Zen Blue 3.8 Software. Adult eyes were imaged with a Zeiss Discovery V12 and processed similarly. Expression for a subset of UAS.human cDNAs was also confirmed in larval brains with 238Y (Aso et al. 2009) and Repo.GAL4 (BL602907) as described (Stinchfield et al. 2024).

### New Tub.GAL80 insertions on FRT101F

Each intergenic region on the fourth chromosome was checked for both endogenous transposable elements and repetitive sequences via JBrowse in FlyBase. Locations lacking both had 1 kilobase (kb) of flanking genomic sequence examined for CRISPR targets containing a 5′ G, the 3 base pair proto-spacer adjacent motif (PAM) sequence NGG at the 3′ end, and no off-target sites. Five locations met the criteria and three were chosen for the insertion of a transgene bearing a tubulin 1alpha promoter ubiquitously driving the yeast GAL4 repressor protein GAL80 (Tub.GAL80; derived from Addgene #17748; Lee and Luo 1999). These regions were in polytene bands 102B (4:405575-405597), 102C (4:489787-489900), and 102F (4:1197868-1197887). Approximately 500 base pairs surrounding each site were amplified from the FRT101F chromosome (Goldsmith et al. 2022) and sequenced to identify any differences from the reference genome. CRISPR homology-directed repair plasmid construction was identical for all three new Tub.GAL80 transgenes and followed Goldsmith et al. (2022). First, a PCR product containing approximately 1 kb distal to the target site (called the 3′ homology arm or homology arm 2 and abbreviated HA2) with 5′ EcoRI and 3′ BamHI sites added, was ligated into the plasmid pUC19 to create pUC19-HA2. The Tub.GAL80 sequence was amplified from Tub.GAL80 in pCaSpeR4 (Addgene #17748). The primers for this PCR added overlapping sequences that matched the pUC19-HA2 plasmid. The entirety of the pUC19-HA2 plasmid was amplified and linearized by PCR to allow for Gibson Assembly of the Tub.GAL80 sequence into pUC19-HA2 just upstream of the HA2 sequence (Gibson et al. 2009). Following construction of pUC19-Tub.GAL80-HA2, the entire sequence was amplified with specific SapI restriction enzyme sites added to the 5′ and 3′ ends of the PCR product for cloning into pHD-DsRed (Homology Dependent Recombination plasmid with an eye-specific promoter driving a variant of Red Fluorescent Protein as a transformation marker; Gratz et al. 2013). Following digestion of the Tub.GAL80-HA2 PCR product and the pHD-DsRed plasmid with SapI, the Tub.GAL80-HA2 PCR product was ligated into pHD-DsRed just distal to the DsRed sequence. This created the pHD-DsRed-Tub.GAL80-HA2 plasmid. The 1 kb genomic sequence proximal to the CRISPR target site that is needed for the 5′ homology arm (homology arm 1 abbreviated HA1) was synthesized by SynBio Tech (Monmouth Junction, NJ) and delivered in the pUC57 plasmid. The sequence was designed to contain AarI restriction enzyme sites on the 5′ and 3′ ends to allow for cloning into the pHD-DsRed-Tub.GAL80-HA2 plasmid. The pUC57-HA1 plasmid was digested with AarI and the HA1 fragment was purified by gel extraction. The pHD-DsRed-Tub.GAL80-HA2 plasmid was then digested with AarI and the HA1 fragment was added, creating the final transgene for CRISPR.

Each gRNA for CRISPR genome editing was provided by a pU6 plasmid (Gratz et al. 2013). gRNAs were created by annealing phosphorylated sense and anti-sense oligos of the desired sequences with overhangs matching a cleaved BbsI restriction site. The pU6 plasmid was digested with BbsI, and the annealed oligos were ligated into place to make the complete pU6-gRNA plasmid. After CRISPR insertion, the entire Tub.GAL80 transgene with the adjoining genomic DNA was sequenced. A complete list of primers for the new Tub.GAL80 stocks is in Supplementary Table 7.

For Cre-dependent removal of DsRed we employed the same crossing scheme with each new Tub.GAL80 stock (Zhang et al. 2014). The insertion in 102B is an example: males who were *TI{TI}FRT101FTI{RFP[DsRed.3×P3.cUa] = Tub-GAL80.W}102B-DsRed + /In(4)ci^D^, ci^D^ pan^ciD^* were crossed to females with *P{y[ + mDint2] = Crey}1b/+; TI{GMR-HMS04515}Gat^eya^/+* (derived from BL766) to introduce Cre recombinase. Progeny with the markers *y^+^* and *Gat^eya^* and without the marker *ci^D^* were sibmated, and their progeny without *y^+^* or DsRed that were *TI{TI}FRT101F TI{Tub-GAL80.W}102B/TI{GMR-HMS04515}Gat^eya^* were sibmated for stocks. The sequence was rechecked across the region after DsRed removal. The new FRT101F Tub.GAL80 stocks, as well as a list of all mutants generated on the FRT101F chromosome, are in Supplementary Table 5.

### FRT101F mutant MARCM clones in eye discs or larval and adult brains

Validation of FRT101F function after CRISPR mutagenesis employed MARCM (Mosaic Analysis with a Repressible Cell Marker; Lee and Luo 1999) clones labeled with GFP in the eye or brain. In brief, *Actin5C.GAL4 UAS.CD8-GFP/CyO; FRT101F mutant/Gat^eya^* males were mated to females homozygous for *heat-shock-FLP; FRT101F-Tub.GAL80-102B*. Twenty-five percent of the progeny will contain the genotype capable of mitotic recombination *Actin5C.GAL4 UAS.CD8-GFP; FRT101F mutant/FRT101F-Tub.GAL80.102B*. For brain clones, eggs were collected in pairs of vials overnight at 25 °C, then aged 48 hours (egg age at heat shock was 48 to 72 hours; during the second larval instar). A one-hour heat shock at 37 °C was applied to one vial, and the eggs were returned to 25 °C until larvae or adult females were collected. The other vial remained at 25 °C as a positive control for GAL80 suppression of GAL4-driven UAS.GFP expression. For eye discs, egg laying was the same, but the heat shock was administered after 24 hours (egg age at heat shock was 24 to 48 hours; during the first larval instar).

For larval brain clones, GFP-positive wandering third instar larvae were picked, dissected, and stained as described (Stinchfield et al. 2024). For adult brain clones, one-day-old adult females were dissected, and GFP-positive brains were fixed and stained as described (Stinchfield et al. 2026). Primary antibodies were mouse α-Repo (DSHB 8D12), α-FasII (DSHB 1D4), α-Coracle (DSHB C615.16), rat α-Shotgun (DSHB DCAD2), and chicken α-GFP (Abcam ab13970). Secondaries were goat α-mouse, α-rat, or α-chicken Alexa Fluor 488 and 633 (Molecular Probes). Brains were mounted in Vectashield antifade media, then imaged on a confocal with 2 µm slices at 20× or.5 µm slices at 100×. For eye discs, GFP detection in clones was the same as 3′-HA detection.

## Results

**Fig. 1.**
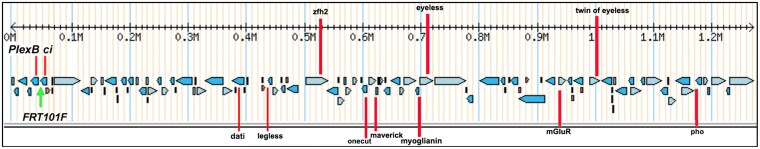
The locations of FRT101F and ten genes with mutant phenotypes described below. The gridline reflects base pair numbering of the fourth chromosome long arm with the centromere to the left. The arm contains 79 protein-coding genes (blue rectangles). The location of FRT101F near the centromere (green arrow), between the genes *PlexB* and *ci*, is shown. Gene and protein expression data are reported for *maverick*. Loss-of-function phenotypes are reported for *datilographo*, *eyeless*, *legless*, *myoglianin,* and *twin of eyeless*. Overexpression phenotypes are reported for human and fly pairs *metabotropic Glutamate Receptor*/GMR1, *onecut*/ONECUT1, *pleiohomeotic*/YY1, and *zfh2*/ZFHX2.

The FCRP has over 850 stocks available at the Bloomington and Kyoto Drosophila Stock Centers (Table 1). A glossary of current genomic technology terms is in Supplementary Table 1. Here we provide illustrations of unexpected mutant phenotypes generated by stocks for ten fourth chromosome genes. A schematic of the fourth chromosome showing the location of each gene is in Fig. 1. We also provide a detailed update of new FCRP stocks, new mutant phenotypes, and results from validation experiments. A complete summary of the FCRP is in Supplementary Table 6. Our goal remains to provide “off the shelf” capability for any investigator to thoroughly analyze any fourth chromosome protein-coding gene.

**Table 1.** Summary of FCRP stocks deposited at Bloomington and Kyoto (869; 12–11–2025).

T2A.GAL4 eGFP	UAS.fly cDNA	UAS.human cDNA	Recombination and FRT101F	Mutations on FRT101F
190	138	175	14	352

### T2A.GAL4 and eGFP data for *maverick* reveal unexpected adult brain expression

The FCRP contribution to the T2A.GAL4 and eGFP stock sets is complete. The expression pattern in the larval brain and ventral cord for new eGFP stocks is in Supplementary Fig. 1. Included is CG11155 (now called *ukar*) that generates two distinct proteins via alternative splicing (Stinchfield et al. 2024). *ukar* encodes one protein in frame zero and two proteins in frame one. eGFP accumulation in the larval brain of the single protein with frame zero is dramatically less than the accumulation of the two proteins with frame two. This dichotomy in expression for the two reading frames was also seen for the other fourth chromosome genes that encode proteins similarly *Asator* and *mGluR* (Stinchfield et al. 2024). For all three genes, the distinction suggests the possibility that the reading frame with reduced expression has a function, distinct from the other reading frame, that is yet to be identified.

Recent efforts focused on converting CRIMIC transgenes that contain a T2A.GAL4 artificial exon to an eGFP artificial exon. The design of the DoubleHeader donor results in the conversion of a CRIMIC T2A.GAL4 transgene to a DoubleHeader T2A.GAL4 or to eGFP at equal rates. Conversion in both cases results in the loss of the 3×P3-GFP transformation marker in the CRIMIC. For DoubleHeader T2A.GAL4 converted genes this opens the green channel for detecting transcription when crossing to UAS.GFP. Detection of transcription in green facilitates side by side comparisons to the gene's eGFP protein accumulation in the same color. Side-by-side comparisons can uncover post-transcriptional regulation such as micro-RNA (e.g. Harsh et al. 2025) or post-translational regulation such as ubiquitylation (e.g. Quasney and Griffith 2025).

Our illustration of CRIMIC DoubleHeader conversion and side by side comparison of the resulting T2A.GAL4 and eGFP stocks employs the enigmatic fourth chromosome gene *maverick* (*mav*). Mav is a secreted Transforming Growth Factor-β family member that is unique in this family. In addition to the family's canonical serine-threonine kinase receptors (Fuentes-Medel et al. 2012), Mav can utilize the Ret tyrosine kinase receptor (Hoyer et al. 2018; Myers et al. 2018). *mav* is also the only Transforming Growth Factor-β protein in flies whose loss of function mutants are homozygous viable and fertile, suggesting a non-essential role. In contrast, the conservation of Mav from nematodes to humans (Wisotzkey and Newfeld 2020), suggests an important function maintained by selection for hundreds of millions of years.

**Fig. 2.**
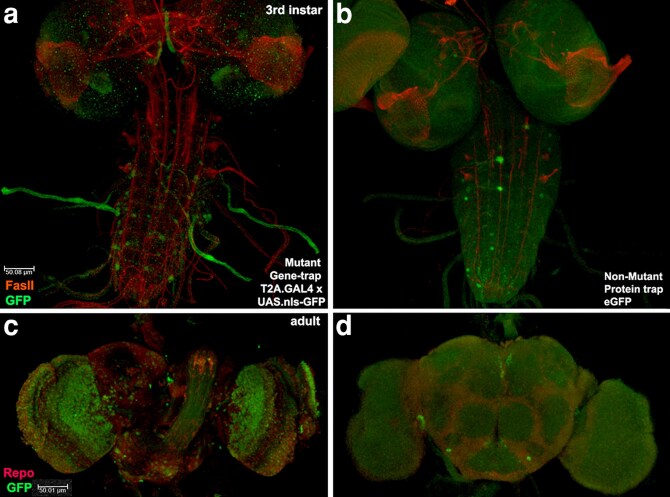
*maverick* brain expression suggests a role in homeostasis. a–d) Larval and adult female brains (*n* = 3 each) reflecting GFP (green) with FasII (red; larvae) or with Repo (red; adults). Left is detection of UAS.nls-GFP driven by *mav* T2A.GAL4 in the *mav* transcription pattern. Right is the detection of eGFP in the endogenous Mav protein. T2A.GAL4 and eGFP alleles of *mav* are homoviable. a) In larval brains, transcription appears confined to a small number of cells in the ventral cord. GFP in tracheal tubes reflects trapped secondary antibody. b) Protein accumulation appears similar. c) In adult brains, extensive transcription is visible in the optic lobes. d) No protein accumulation is visible in adult brains, suggesting either efficient secretion or post-transcriptional regulation. An update of new T2A.GAL4 and eGFP stocks is in Supplementary Table 2.

Given these conflicting circumstances, we hypothesized that *mav* played a vital role in adult homeostasis. For the hypothesis test, we examined *mav* transcription via DoubleHeader converted T2A.GAL4 in a side-by-side comparison to Mav eGFP protein accumulation in the adult brain (Fig. 2). The comparison revealed an unexpectedly large amount of transcription with diffuse protein accumulation, indicating efficient secretion. We then examined *mav* T2A.GAL4 mutant adult longevity in comparison to adults with the parental genotype (*y w*) as a control for genetic background (Table 2). The data showed that *mav* mutant flies have significantly shorter lives (*P* < 0.05) in both sexes and their lives are shorter as either unmated or mated individuals. Reduced lifespan with extensive brain expression suggests *mav* has a role in the brain during adult homeostasis influencing lifespan. This role is one possible means of reconciling the viability of *mav* mutants with is millennia of evolutionary conservation.

**Table 2.** Lifespan of mav T2A.GAL4 males and females (as virgins and after mating) is significantly shorter than their parental stocks.

Genotype	Mean lifespan	*P*-value vs *yw*	Genotype	Mean lifespan	*P*-value vs *yw*
*yw* virgin female	46.4 ± 14.3		*yw* mated female	50.5 ± 16.7	
*mavT2A* virgin female	40.1 ± 11.6	0.04	*mavT2A* mated female	35.7 ± 14.2	<0.01
*yw* virgin male	58.0 ± 16.5		*yw* mated male	56.7 ± 17.6 7.616.7	
*mavT2A* virgin male	33.9 ± 12.4	<0.01	*mavT2A* mated male	35.7 ± 11.9	<0.01

### Eye expression of UAS fly cDNAs revealed unexpected differences in protein stability

The FCRP contribution to the sets of 3′-HA tagged UAS fly and UAS human cDNA stocks is nearly complete. Note, we do not tag fly or human cDNAs encoding proteins with data showing a carboxy-terminal tag interferes with function (e.g. Transforming Growth Factor-β proteins; Entchev et al. 2000). Recent efforts employ the developing eye to validate UAS fly and UAS human cDNA stocks by driving them with GMR.GAL4 in differentiating cells during the third larval instar. Expression of GMR.GAL4 initiates in all cells starting approximately four rows behind the advancing morphogenetic furrow. The GAL4 driver remains on throughout the posterior domain of the eye disc. These experiments allow verification of 3′-HA expression for those with tags and assessment of potential function if a mutant phenotype is generated.

All FCRP UAS fly cDNA stocks with a 3′-HA tag in our standard vector (pGW-HA.attB; Stinchfield et al. 2024) display expression in eye discs except Kif3C (Supplementary Fig. 2 and Supplementary Table 4). The explanation is that Kif3C is normally repressed at the mRNA level by *miR-184* (Iovino et al. 2009). On the other hand, UAS fly cDNA stocks with a 3′-HA tag in the legacy UFO vector have no detectable HA expression. This is one of the problems we detected through validation experiments. The solution was to rebuild these UAS fly cDNA stocks in pGW-HA.attB to replace the non-expressing stocks at Bloomington and Kyoto. All FCRP UAS human cDNA stocks with a 3′-HA tag show expression in eye discs except DYRK1 (Supplementary Fig. 3 and Supplementary Table 4). A stock with this gene is in the process of being rebuilt to replace the non-expressing one.

The image data also reveals noticeable differences in protein stability among the fourth chromosome proteins. For example, CamKI, CG1674, CG1909, Dati, Lgs, and ND-49 are highly expressed throughout the GMR.GAL4 spatial pattern (Supplementary Fig. 2), implying that they are stable. In contrast, the expression of 4E-T, CG31988, and Gat is much lower, suggesting that these proteins may be unstable. From a different perspective, we noted that expression of CG33521, Pho, and PMCA, while high, ends a few rows after the GMR.GAL4 pattern initiates. This could suggest that these proteins are subject to post-transcriptional regulation or are actively degraded within particular spatial domains.

Similar trends are observed with the orthologous human 3′-HA tagged cDNAs. For example, TRIP6 (Human Genome ID 12311) is highly expressed and distributed throughout the GMR.GAL4 spatial pattern (Supplementary Fig. 3). In contrast, ZFHX2 (ID 20152) is highly expressed but extinguished a few rows after initiation. A further contrast is seen with RNF6 (ID 10069), which is both weakly expressed and rapidly extinguished. Taken together, the eye disk data show that examination of tagged cDNA transgenes provides a rapid means for identifying proteins experiencing post-transcriptional regulation.

### Eye expression of human neurological genes YY1 and GRM1 suggests new disease models

**Fig. 3.**
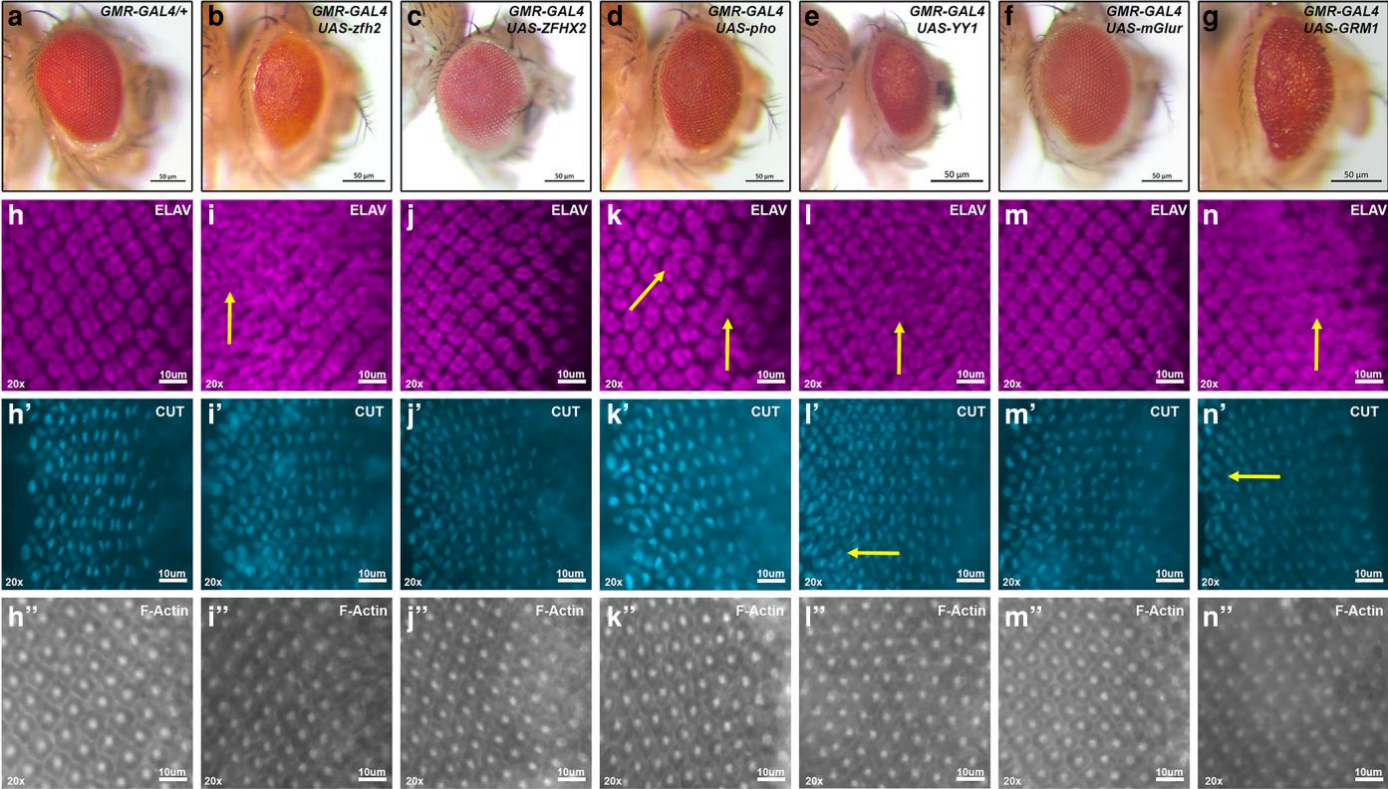
Human neurological disease genes YY1 and GRM1 induce a rough eye phenotype. a–g) Adult eyes anterior to the right. a) GMR.GAL4 heterozygous control. b,c) Expression of fly Zfh2 results in a rough eye phenotype, while human ZFHX2 has no effect. d,e) Expression of fly Pho and human YY1 causes varying degrees of roughness, with Pho having a weaker effect than YY1. f,g) Expression of fly mGluR has no effect, while expression of human GRM1 causes a severe rough eye. h–n) Larval eye-antennal discs. h) Wild-type pattern of photoreceptors expressing Elav. h′) Cone cells expressing Cut. h″) Ommatidial clusters expressing F-Actin. i–i″) Severe disorganization of photoreceptors (yellow arrow) and ommatidial clusters with Zfh2, but no effect in cone cells. j–j″) No defects with ZFHX2. k–k″) Pho shows mild disorganization of photoreceptor columns (yellow arrows) and cone cells. l–l″) YY1 shows a loss of compaction of photoreceptors into clusters (yellow arrow), the presence of extra cone cells (yellow arrow), and disorganized ommatidia. m–m″) mGluR has no effect. n–n″) GRM1 shows severe disruption of photoreceptors (yellow arrow), but no effect on cone cells or the ommatidial pattern. An update of new UAS fly and human cDNA stocks is in Supplementary Table 3 and a summary of UAS fly and human cDNA 3′-HA tag eye disc expression and adult eye phenotypes is in Supplementary Table 4.

If a GMR.GAL4-driven cDNA has an impact on eye development, and this may be apparent in the adult eye. Rough eyes are often smaller than wild type with a distinct “pebbled” look of mismatched rows across the surface. Glazed eyes produce a smooth “glossy” appearance that can be accompanied by the retention or loss of pigment. The presence of pigment indicates that the cone cells are reduced/lost while the pigment cell array is maintained. The loss of pigment indicates the retention of cone cells but the reduction/loss of pigment cells. The differences in the pigment of glazed eyes can be used to discern how development has been derailed. Examination of adult eyes identified three orthologous pairs of human and fly genes where at least one of the stocks yielded a rough eye (Fig. 3). In the first pair, fly Zinc finger homeodomain 2 (Zfh2) is a transcription factor not normally present in the eye (Raja et al. 2024). Expression of Zfh2 produces a rough eye with eye discs showing disorganization and compaction of photoreceptor clusters, but unaffected cone cells. Expression of human ZFHX2 and ZFHX3 (ID 777) was validated with HA detection in discs but had no effect on the eye.

The second pair is fly Pleiohomeotic (Pho) and human YY1 (ID 12856). Pho is a transcriptional repressor impacting chromatin via interactions with the Polycomb group of proteins. Pho knockdown by RNAi had no effect on the eye (Brown et al. 2023). YY1 functions as both a transcriptional activator or repressor via interactions with multiple chromatin proteins, including Polycomb (Verheul et al. 2020). YY1 deletions are the cause of autosomal dominant Gabriele deVries syndrome (Gabriele et al. 2017). How the loss of YY1 leads to cognitive impairment and a wide spectrum of morphological abnormalities visible at birth is unknown. We found that expression of Pho or YY1 leads to a rough eye, with YY1 having a stronger effect. In Pho eye discs, the rows of photoreceptor clusters are slightly disorganized with defects in both photoreceptor and cone cell numbers. Many ommatidia have fewer than the normal complement of both cell types. In YYI discs, there are no evenly spaced rows of photoreceptor clusters and the clusters themselves appear incomplete. In addition, there are numerous extra cone cells, although the overall organization of the ommatidia is normal. One hypothesis is that a subset of photoreceptor neurons shifted to the glial cone cell fate. Another possibility is that the loss of photoreceptors is coupled to a separate but independent gain of cone cells. Analysis of the YY1 mechanism of action in the eye could serve as a model for revealing how haploinsufficiency leads to the multifactorial symptoms of Gabriele deVries syndrome.

The third pair is the fly metabotropic Glutamate Receptor (mGluR) and human GRM1 (ID 4593). Both are G-protein coupled receptors for the excitatory neurotransmitter glutamate. mGluR is not present in the eye (Raja et al. 2024) and has no effect when driven by GMR.GAL4 (while not HA tagged, a phenotype due to expression from UAS.mGluR was reported; Pan and Broadie 2007). Alternatively, GRM1 causes a severe rough eye. GRM1 deletions are the cause of autosomal dominant spinocerebellar ataxia 44. GRM1 missense mutations are the cause of autosomal recessive spinocerebellar ataxia 13 (Protasova et al. 2023). Like Gabriele deVries syndrome, both ataxias have overt physical symptoms as well as cognitive impairment and developmental delay that are visible at birth. How haploinsufficiency of GRM1 leads to ataxia is unknown. In GRM1 eye discs, photoreceptor clusters appear overgrown, thus eliminating normal spacing, while cone cells and ommatidial organization are normal. One hypothesis is that a subset of photoreceptor neuron precursors experienced an additional cell division. Analysis of the GRM1 mechanism of action in the eye could serve as a model for understanding its two ataxias.

**Fig. 4.**
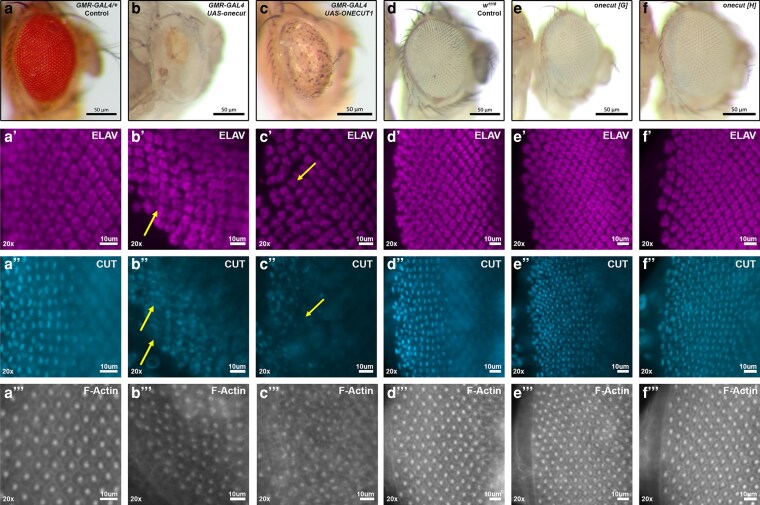
Fly and human Onecut induce a glazed eye phenotype but *onecut* mutations do not. a–f) Adult eyes anterior to the right. a) GMR.GAL4 heterozygous control. b,c) Expression of fly Onecut or human ONECUT1 result in a severe glazed eye. d) *white* eye control. e,f) Eyes from adults homozygous for either *onecut^G^* or *onecut^H^* show no defects. a′) Wild type pattern of photoreceptors expressing Elav. a″) Cone cells expressing Cut. a‴) Ommatidial clusters expressing F-Actin. b′–b‴) Expression of fly Onecut leads to severe disorganization of photoreceptors (yellow arrow), supernumerary cone cells (yellow arrows) and ommatidial disorganization. c′–c‴) Expression of human ONECUT1 disrupts photoreceptor spacing, leads to loss of cone cells and increased ommatidial disorganization. d′–d‴) Wild type patterns for the three markers. e′–e‴) *onecut^G^* homozygosity does not impact any marker. f′–f‴) *onecut^H^* homozygosity does not impact any marker.

Fly Onecut and human ONECUT1 (ID 8138; previously known as Hepatocyte nuclear factor6 and associated with autosomal dominant polycystic liver disease) were the only orthologous pair to generate a glazed eye (Fig. 4). Both encode transcription factors with two DNA-binding domains (Kropp and Gannon 2016). Fly Onecut was reported to function in the eye to maintain photoreceptor cell fate based on overexpression of a chimeric dominant negative transgene (Nguyen et al. 2000). At the time, there were no mutations in *onecut*. In the mouse retina, ONECUT1 functions differently to specify ganglion and horizontal cells (Sapkota et al. 2014). Examination of eye discs expressing either fly or human Onecut showed flawed photoreceptor and cone cell specification, resulting in defective organization of the ommatidial rows (Supplementary Fig. 4). Onecut eGFP extended prior RNA in situ expression data by showing that protein accumulation begins after the final phase of R8 specification but prior to the specification of the R3/R4 photoreceptors. Homozygosity for either of two FCRP *onecut* truncation alleles (Weasner et al. 2025) has no effect on the eye disc or adult eye. The data suggest that while fly Onecut has a redundant role in photoreceptor maintenance, human ONECUT1 has a distinct function in the mammalian retina.

As a next step for the three human genes with disease associations that generate eye phenotypes, it seems fruitful to apply the full battery of techniques deployed by the Model Organism Screening Centers of the Undiagnosed Diseases Network (Yamamoto et al. 2024). These methods are often able to quickly identify a human gene's mechanism of action.

### New Tub.GAL80 transgenes and mutants on the FRT101F chromosome for MARCM

Roughly three years after the creation of the FRT101F Tub.GAL80-101F chromosome (Goldsmith et al. 2022), we noticed Actin5C.GAL4-driven UAS.GFP expression in the presence of Tub.GAL80 in a subset of tissues. The suppression of GAL80 expression leading to unregulated Actin5C.GAL4-driven UAS.GFP prevents these chromosomes from effectively generating marked mutant single-cell clones (MARCM) for the analysis of a fourth chromosome gene. The most prominent problem was in imaginal discs, where MARCM is a very common means of analysis. Other tissues, such as larval and adult brains, showed no problems. We concluded that the combination of proximity to centromeric heterochromatin and insertion into an endogenous 1,360 transposable element capable of locally nucleating heterochromatin (Sun et al. 2004), over time, led to heterochromatization of Tub.GAL80-101F. The presence of heterochromatin reduced the ability of the tubulin promoter to activate transcription of GAL80, leading to reduced GAL80 expression and intermittent suppression of Actin5C.GAL4-driven UAS.GFP. The adjacent FRT101F retains its ability to recombine, as shown by the capacity to make clones in larval discs and brains, as well as adult brains. The Tub.GAL80-101F stocks (with and without DsRed) were withdrawn from the stock centers, and we generated stocks with new Tub.GAL80 insertions on the FRT101F chromosome.

**Fig. 5.**
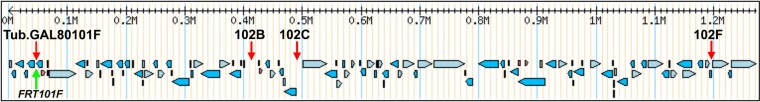
Original and three new Tub.GAL80 transgenes on the FRT101F chromosome. Fourth chromosome is displayed as in Fig. 1. The original Tub.GAL80-101F was inserted immediately distal to FRT101F as indicated with a red arrow at the far left. These were placed between *PlexB* and *ci* after base pair 46,995 (Goldsmith et al. 2022). The new Tub.GAL80-102B is between *dati* and *lgs* after base pair 405,575. The new Tub.GAL80-102C is between *Asator* and *zfh2* after base pair 489,878. The new Tub.GAL80-102F is between *PIP4K* and *Mitf* after base pair 1,197,868. Details of each new insertion site (red arrows) are shown in Supplementary Fig. 5. Evidence of complete suppression of Actin5C.GAL4 driven UAS.GFP by the new Tub.GAL80 transgenes in 102B and 102C (similar to the parental Tub.GAL80 on the X chromosome; BL42726) are shown in Supplementary Fig. 6. Also shown is variegating Actin5C.GAL4-driven UAS.GFP with Tub.GAL80-101F and Tub.GAL80-102F.

In designing the new insertions, we realized that placement in regions devoid of endogenous transposable elements and repeat sequences was essential. Regions were chosen in polytene bands 102B, 102C, and 102F. The locations of all four Tub.GAL80 insertions on the FRT101F chromosome are in Fig. 5 (see Supplementary Fig. 5 for details). The three new Tub.GAL80 transgenes were initially shown to suppress Actin5C.GAL4-driven UAS.GFP in the eye disc. However, shortly thereafter Tub.GAL80-102F began losing its ability to suppress Actin5C.GAL4-driven UAS.GFP in the eye disc. Tub.GAL80-102B and Tub.GAL80-102C remain strong suppressors of Actin5C.GAL4-driven UAS.GFP in the eye, wing, and leg discs (Supplementary Fig. 6) as well as larval and adult brains (discussed below).

Both Tub.GAL80-102B and Tub.GAL80-102C stocks are available from Bloomington and Kyoto (Supplementary Table 5a) with the DsRed transformation marker present allowing for tracking of the Tub.GAL80 transgene in crossing schemes or with DsRed removed to open another wavelength for analysis (we visualize DsRed in the blue channel at 546 nm). Our qualitative assessment is that centromere and telomere heterochromatin spreading decreased the expression of the Tub.GAL80 insertions at 101F and 102F. Our advice to colleagues before attempting MARCM on the fourth chromosome is to cross Actin5C.GAL4 with UAS.GFP to one of the FRT101F Tub.GAL80 chromosomes. Then, examine your tissue of interest in progeny to ensure that GFP is suppressed.

FCRP has continued to mutagenize protein-coding genes on the FRT101F chromosome for use with the new Tub.GAL80 chromosomes in MARCM. Our recent paper (Weasner et al. 2025) provides details of our mutagenesis methods, mutant sequences, lethality of new mutations over a deletion, and tests for second-site mutations. Supplementary Table 5 contains a complete list of stocks with mutations on the FRT101F chromosome, updating the list in Weasner et al. (2025). It is now possible to examine the phenotype elicited by a loss of function mutation for every protein-coding gene on the fourth chromosome at the organismal level and for 96% of these genes at the single-cell level via MARCM.

### Mutant MARCM clones reveal new phenotypes for unstudied and well-studied genes

**Fig. 6.**
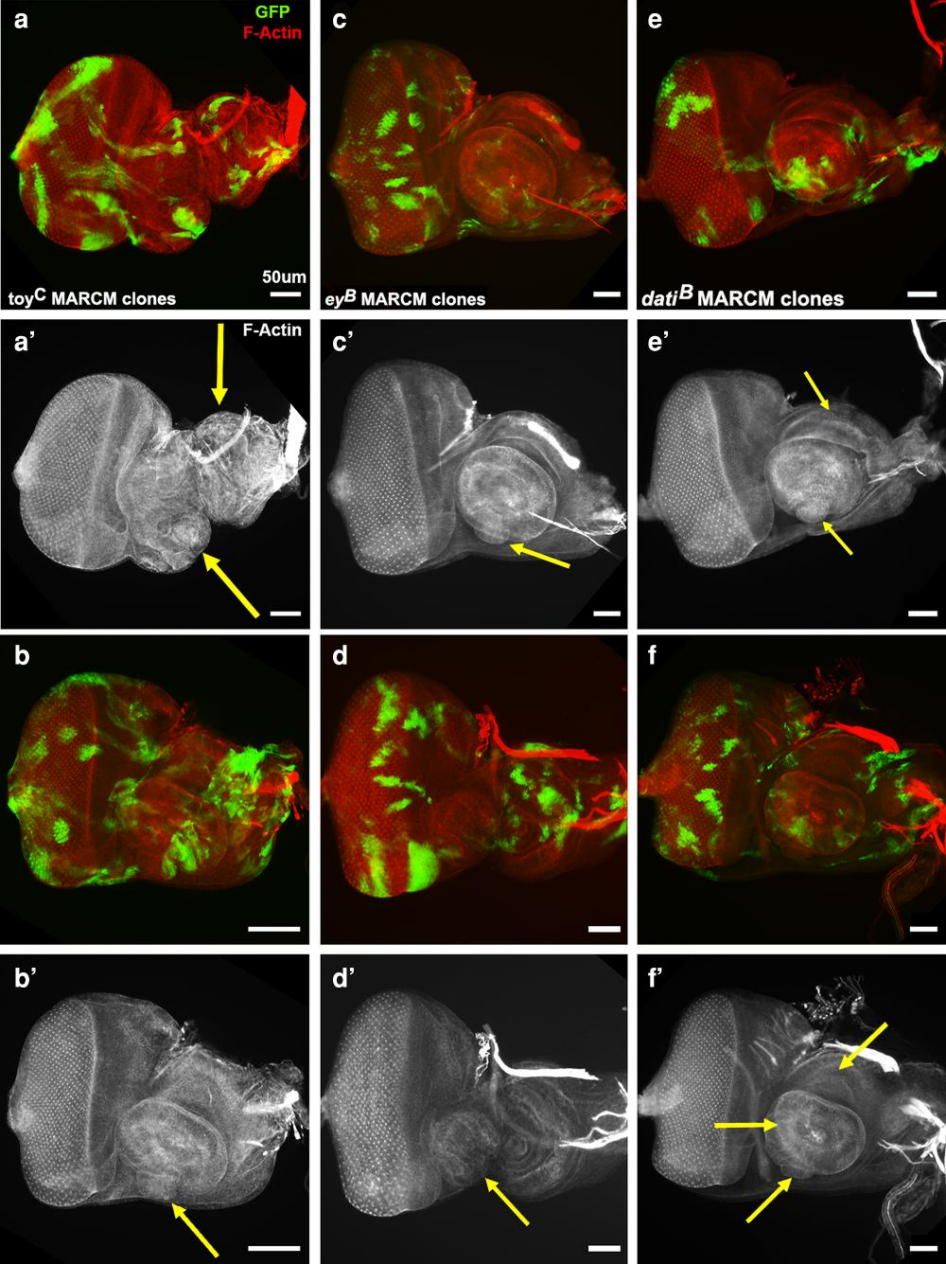
*toy^C^*, *ey^B^*, and *dati^B^* MARCM clones induce antennal duplications in larval eye-antennal discs. Third instar discs from wandering larvae subjected to a one-hour heat shock while 0 to 48 hours old are shown in two colors or F-actin alone. Homozygous mutant clones are marked with GFP. a,b) Two discs with antennal duplications (yellow arrows) associated with *toy^C^* clones. The top disc has two duplications. c,d) Two discs with antennal duplications (yellow arrows) associated with *ey^B^* clones. e,f) Two discs with antennal duplications (yellow arrows) associated with *dati^B^* clones. Both discs have multiple duplications.

To date, 16 FRT101F mutant chromosomes have been validated with MARCM clones in larval eye discs or the brain (Supplementary Table 6; validated allele indicated). For example, three transcription factors, including the Paired Box proteins *eyeless* (*ey*) and *twin of eyeless* (*toy*), as well as the zinc finger protein *datilographo* (*dati*) were validated in the eye disc using Tub.GAL80-102C (Fig. 6). *ey* and *toy* have well-established and conserved roles during eye development (Kumar 2009). Alternatively, *dati* is effectively unknown with no detailed studies and is mentioned in just a few genome-wide studies. Prior to the FCRP, the only known *dati* mutation was a MiMIC insertion in an exon that is homoviable and has never been reported in a paper. We generated MARCM clones of the homozygous lethal truncation alleles *ey^B^* (stop after amino acid 291 of 898), *toy^C^* (stop after amino acid 74 of 655) and *dati^B^* (stop after amino acid 395 of 1,150). We find that clones of all three mutations can result in antennal disc duplications or defects. This effect is stronger in *toy^C^* and *dati^B^* clones than in *ey^B^* clones. The duplications appear to be both cell autonomous and non-autonomous. A past study showed that blocking apoptosis in *ey* loss-of-function mutants results in antennal duplications (Punzo et al. 2004). Perhaps in all three transcription factor mutant clones, apoptosis is blocked, thus inducing antennal duplication? Or perhaps *dati^B^* induces duplications via a mechanism independent of *ey* and *toy*? For *ey* and *toy*, these results move beyond a previous report that identified the same phenotype with flip-out RNAi clones (Zhu et al. 2017). The advance is that the MARCM clones show antennal duplication is not a synergistic effect of *ey* and *toy* simultaneous loss, but instead a redundant function with *ey* and *toy* fulfilling the same role. For *dati*, the antennal duplication phenotype opens a window of opportunity for studying a gene whose role in development and adult homeostasis is completely unknown.

**Fig. 7.**
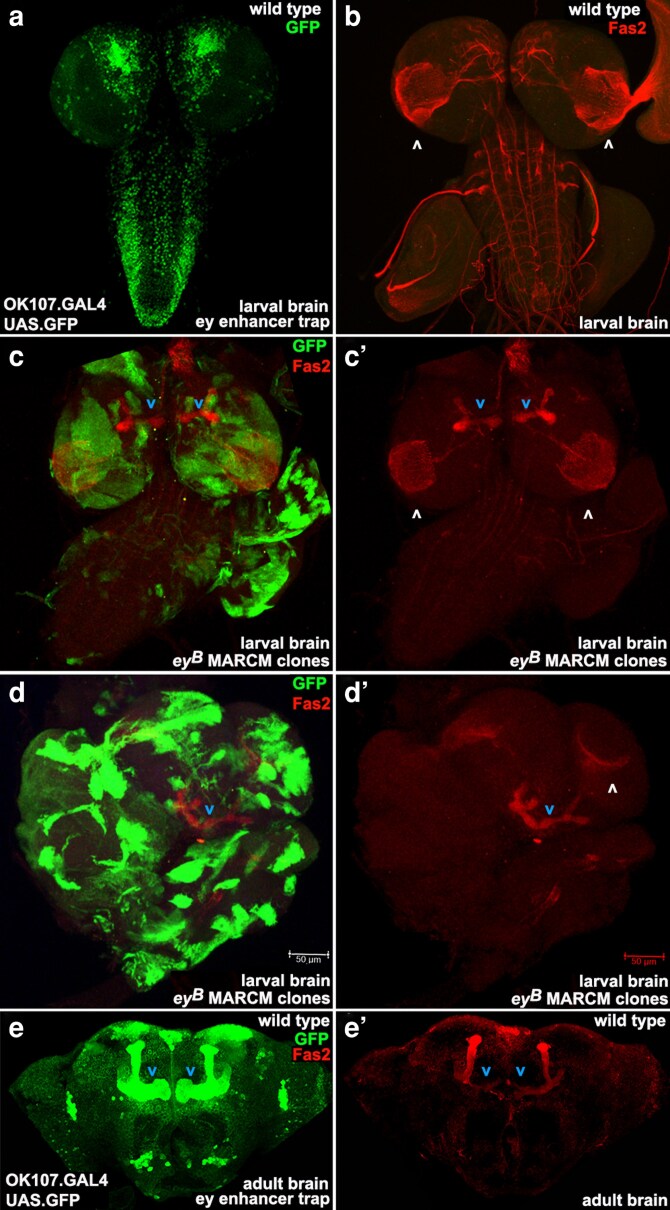
*ey^B^* clones induce a heterochronic phenotype with an adult-shaped mushroom body in a larval brain. a) Wild-type larval brain with *ey* expression via OK107.GAL4-driven UAS.GFP in bilaterally symmetrical Kenyon cells of the mushroom body at the top of the brain. b) Wild-type larval brain with FasII in mature neurons of the optic lobe (white arrowheads), central brain, and ventral cord, but not in the mushroom body. c) Larval brain with multiple GFP-marked *ey^B^* clones displays FasII in a nearly normal-shaped adult mushroom body (blue arrowheads). c′) The same brain shows FasII is normal in the optic lobe (white arrowheads) and present in a nearly normal-shaped adult mushroom body (blue arrowheads). d) Highly deformed larval brain with multiple GFP-marked *ey^B^* clones and FasII in a distorted but recognizable adult-shaped mushroom body single lobe (blue arrowhead). d′) The same brain shows FasII has expression in one optic lobe (white arrowhead) and in the distorted but recognizable adult-shaped mushroom body single lobe (blue arrowhead). e) Wild-type adult brain displaying *ey* expression via OK107.GAL4-driven UAS.GFP in the bilaterally symmetrical, L-shaped mushroom body (blue arrowheads). e′) The same adult brain shows FasII in the bilaterally symmetrical, L-shaped mushroom body (blue arrowheads).

Beyond the larval stage, *ey* has prominent adult expression in two brain regions, insulin producing cells and mushroom body neurons, as seen with the enhancer trap OK107.GAL4 (Tran et al. 2018). During embryonic and larval stages, *ey* regulates *dilp5* expression (Okamoto et al. 2012) and maintains mushroom body neuroblast cell cycle independence from nutrient limitation (Sipe and Siegrist 2017). However, *ey* roles in larval mushroom body neuroblasts have not been connected to neuronal differentiation. This is in contrast with the larval optic lobes, where *ey* is a medulla neuroblast temporal transcription factor that regulates neuronal fate (Li et al. 2013). To determine if *ey* has a role in neuronal differentiation in the larval mushroom body, we induced MARCM clones with Tub.GAL80-102B during the second instar. We examined expression of FasII, a well-established membrane marker for mature neurons in the mushroom body and beyond. Unexpectedly, *ey^B^* clones induced a heterochronic phenotype with a FasII expressing adult shaped mushroom body in the larval brain. While not completely wild type, we show two examples of brains with *ey^B^* clones that contain recognizable FasII expression in an L-shaped, adult mushroom body (Fig. 7). While well known in nematodes (e.g. Liu and Ambros 1989) we could find no reports of a heterochronic phenotype in flies. Further, in contrast to medulla neuroblasts, where loss of *ey* blocks neuronal differentiation, in mushroom body neuroblasts, loss of *ey* appears to accelerate differentiation, suggesting a repressor rather than an activator role.

**Fig. 8.**
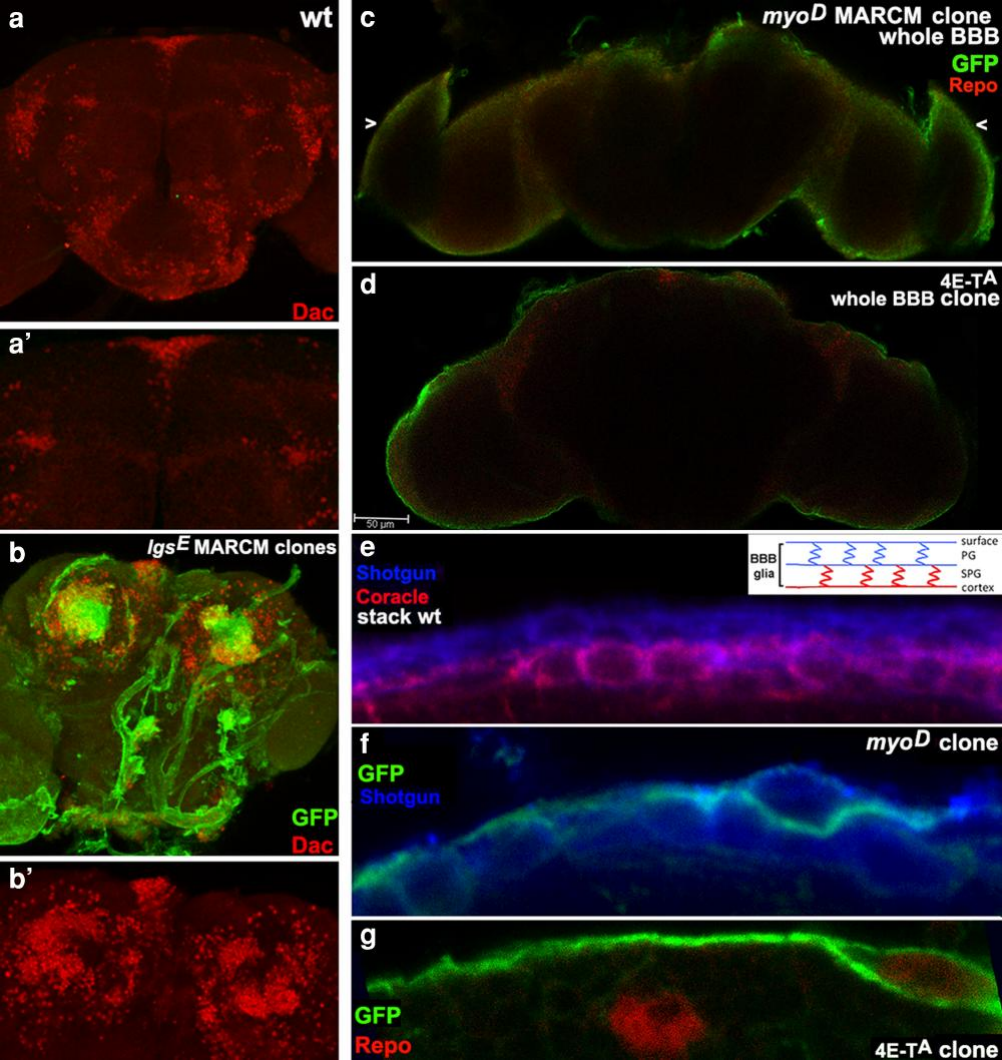
*lgs^E^* and *myo^D^* clones in adult brains reveal roles as repressors of gene expression and growth. One-day-old brains were subjected to a one-hour heat shock during the second instar. a,a′) Dac (red) is normally absent from the medial regions of the central brain. b,b′) Brain with GFP-marked homozygous clones of *lgs^E^*. Clones of *lgs^E^* in medial regions of the central brain are associated with autonomous and non-autonomous expression of Dac. c) Brain with GFP-marked homozygous clone of *myo^D^* that occupies the entire blood-brain barrier is associated with overgrowth in the underlying optic lobes (arrowheads). d) Brain with GFP-marked homozygous clone of *4E-T^A^* that occupies the entire blood-brain barrier has normal optic lobes. e) High magnification view of the two-layer blood-brain barrier in a one-day-old adult brain with Shotgun (blue; adherens junctions) in both layers and Coracle (red; septate junctions analogous to mammalian tight junctions) in the medial subperineural layer. f) High magnification view of the *myo^D^* complete blood-brain barrier clone that occupies the subperineural layer. g) High magnification view of the *4E-T^A^* complete blood-brain barrier clone that occupies the apical perineural layer (Repo in red marks glial nuclei).

Additional mutations on the FRT101F chromosome were analyzed with MARCM clones in the adult brain employing Tub.GAL80-102B (Fig. 8). One mutation is in *legless* (*lgs*), an essential component of Wingless signal transduction (van Tienen et al. 2017). MARCM clones of *lgs^E^* in medial regions of the central brain elicited both autonomous and non-autonomous Dachshund (Dac) expression. Wingless signaling initiates the expression of secreted ligands at various times during development (e.g. leg discs, Estella et al. 2012), potentially explaining non-autonomous Dac expression. To date, the only reported role for Wingless in the adult brain is in the mushroom body, where it contributes to long-term memory (Tan et al. 2013). The *lgs^E^* clone phenotype suggests Wingless has additional roles in adult brain homeostasis.

A second mutation is in *myoglianin*, a conserved member of the Transforming Growth Factor-β family of secreted signaling proteins. We easily generated MARCM clones of *myo^D^* that encompass the entire glial blood-brain barrier (i.e. the clone completely covers the brain). *myo* is normally expressed in a subset of glia in both layers of the larval and adult blood-brain barrier (Stinchfield et al. 2026). Its function there is unknown. Our clone data shows that the loss of *myo* throughout the blood-brain barrier, starting in the second instar, induces optic lobe overgrowth. A closer look at multiple clones revealed that *myo^D^* complete blood-brain barrier clones occur either in the apical perineural layer or the medial subperineural layer but not both, consistent with prior reports that each layer forms independently (Awasaki et al. 2008). The loss of *myo* in either layer has the same effect on the optic lobe.

When inducing clones during the second larval instar, entire blood-brain barrier clones were obtained for *4E-T^A^*, *Kif3C^C^*, and others. These total barrier clones had no obvious effect on the brain. The ability to make mutant clones in the entire blood-brain barrier provides unprecedented opportunities to explore the basic biology of the barrier's selective permeability and to test the barrier-crossing ability of therapeutics for brain disorders. The ease with which interesting phenotypes are elicited by clones of fourth chromosome genes was itself unexpected, but we hope it will encourage investigators to look for themselves.

### FCRP stock update

At the Bloomington and Kyoto stock centers, you can find most FCRP stocks with a search of “Fourth Resource” on the home page. In addition, at Bloomington, there is a dedicated page for FCRP stocks accessed by clicking “More” under Browse Stocks on the home page and then “More Stocks” on the next page. The fifth line down is “Fourth Chromosome Project” that takes you to bdsc.indiana.edu/stocks/misc/fourth_chr.html.

In Supplementary Info we provide an update of all stocks deposited since Stinchfield et al. (2024). The list of new genetic conversions of the fourth chromosome MiMIC and CRIMIC transgenes into T2A.GAL4 gene traps and eGFP protein traps are in Supplementary Table 2. The 3′-HA tagged UAS fly and UAS human cDNAs for protein coding genes in attP sites on chromosome two or three are in Supplementary Table 3. Images of our validation experiments for 3′-HA expression in eye discs of UAS fly and UAS human cDNA stocks are in Supplementary Figs. 2 and 3. A summary of expression for 3′-HA tagged UAS fly cDNA and UAS human cDNA stocks is in Supplementary Table 4. FRT101F chromosomes with a Tub.GAL80 insertion in either 102B or 102C and CRISPR mutations in protein-coding genes are in Supplementary Table 5. A complete summary of FCRP stocks available for fourth chromosome protein-coding genes is in Supplementary Table 6.

To date, 98% of 79 protein-coding genes have a T2A.GAL4 gene trap and 82% of genes have an eGFP protein trap. So far, 92% genes have at least one UAS fly cDNA stock, while 93% of the conserved genes have at least one UAS human cDNA stock. FCRP has mutagenized 96% of genes on the FRT101F chromosome. Many genes have two or three mutations with one or two truncations plus an internal deletion. Note that only truncation alleles are available from Bloomington, while all FRT101F mutagenized stocks, including internal deletions, are available from Kyoto. To date, over 1,000 FCRP stocks have been shipped from the two stock centers.

## Discussion

The FCRP is an effort to provide tools for analyzing genes on the fourth chromosome as if they were on any other chromosome. An example of the utility of FCPR stocks is the application of T2A.GAL4, eGFP, and UAS fly cDNA stocks in an analysis of *apolipophorin* in the adult brain (Stinchfield et al. 2026). We continue to bolster confidence in our stocks through validation. We recently reported in Weasner et al. (2025), heterozygous lethality over a deletion for mutations generated on the FRT101F chromosome. In Stinchfield et al. (2024), we reported the homozygous lethality of T2A.GAL4 and eGFP stocks. Here we note the expression of 3′-HA tags for UAS fly and human cDNAs. We also describe UAS cDNA expression in adult eyes to identify any visible phenotypes. The straightforward data generated by these validation studies should assist in overcoming the notorious reputation of the fourth chromosome as a difficult place to work. In response to community inquiries, the FCRP recently supported publication of a simple remedy for fourth trisomy, one of the most common problems in fourth chromosome genetics (Cook 2025).

Illustrating the utility of FCRP stocks revealed numerous unexpected mutant phenotypes. Studies of expression and MARCM clones in adult brains for *myo* and *mav* indicated that both likely have functions in adult homeostasis. Examination of MARCM clones for *ey* showed distinctive phenotypes in both the larval eye disc and brain. In the eye disc, *ey* clones revealed an overlapping role with its paralog *toy* in antenna formation; the loss of either gene leads to duplications. Antennal duplications were also elicited by mutant clones of the previously unstudied gene *dati,* providing a convenient entry point for further investigation. In the larval brain, mutant clones of *ey* resulted in a unique heterochronic phenotype displaying an adult-shaped mushroom body. Timed transcription factor expression is well known as an activator of neuronal cell fate pathways (Holguera and Desplan 2018). This heterochronic phenotype reveals that temporal transcription factors can also play repressor roles, with loss of *ey* leading to acceleration of mushroom body neuron maturation. Also in the adult brain, a previously unknown role for Wingless as a repressor was revealed through mutant clones of its signal transducer *lgs* that engendered extensive ectopic Dac.

Overexpression studies in the eye with three human cDNAs, each associated with a different autosomal dominant disease, generated relatively straightforward phenotypes. Further experiments that reveal the molecular basis of the phenotypes are likely to shed light on how defective versions of the human genes cause disease. This approach has been applied repeatedly by the Undiagnosed Disease Network, though not to any fourth chromosome genes. UDN studies of human genes in flies make crucial contributions toward diagnosis and treatment of patients (e.g. Chung et al. 2020, 2022).

Currently, the FCRP is working to complete the set of fourth chromosome UAS.RNAi stocks began by the Transgenic RNAi Project (Perkins et al. 2015). We will generate stocks for genes that have no RNAi stocks, or were constructed in the problematic Valium10. For existing RNAi stocks, we evaluate their knockdown effectiveness and detect off-target effects via comparative stage of lethality tests when ubiquitously expressed with a T2A.GAL4 mutation in the same gene. The expectation is that the lethal stage of a ubiquitously expressed UAS.RNAi transgene will match the lethal stage of the gene's T2A.GAL4 stock and that ubiquitous UAS.RNAi will not be lethal for a non-lethal T2A.GAL4 stock. For T2A.GAL4 mutations with neither homozygous lethality nor a visible phenotype, we will employ qPCR to identify the existing UAS.RNAi stock with the quantitatively strongest knockdown of the target mRNA. This data allows investigators to determine which RNAi stock is best when studying a gene that has multiple available stocks.

Overall, the goal of the FCRP is to provide stocks that allow anyone to study a gene on the fourth chromosome as easily as on any other chromosome. In addition to generating stocks, the FCRP provides confidence through validation and an incentive via phenotyping to tackle the many genes on the fourth chromosome that have long been ignored. FCRP stocks facilitate a myriad of biochemical, genetic, and molecular analyses. The stocks are readily available to enhance our understanding of metazoan biology, including the creation of new models for human disease.

## Supplementary Material

jkag077_Supplementary_Data

## Data Availability

The authors affirm that all data necessary for confirming our conclusions are present in this article, figures, tables, Supplementary information, or publicly available databases such as FlyBase (Öztürk-Çolak et al. 2024) and FlyPush (flypush.research.bcm.edu). Stocks are available at the Bloomington Drosophila Stock Center (bdsc.indiana.edu) and the Kyoto Drosophila Stock Center (dgrc.kit.ac.jp) with RRID numbers for both locations in Supplementary information. Technical details for the construction of an individual stock are available upon request. Supplemental material available at G3 online.

## References

[jkag077-B1] Aso Y et al 2009. The mushroom body of adult Drosophila characterized by GAL4 drivers. J Neurogenet. 23:156–172. 10.1080/01677060802471718. 19140035

[jkag077-B2] Awasaki T, Lai SL, Ito K, Lee TJ. 2008. Organization and postembryonic development of glial cells in the adult central brain of Drosophila. J Neurosci. 28:13742–13753. 10.1523/JNEUROSCI.4844-08.2008. 19091965 PMC6671902

[jkag077-B3] Bischof J et al 2013. A versatile platform for a comprehensive UAS-ORFeome library in Drosophila. Development. 140:2434–2442. 10.1242/dev.088757. 23637332

[jkag077-B4] Bischof J, Maeda RK, Hediger M, Karch F, Basler K. 2007. An optimized transgenesis system for Drosophila using germline-specific phiC31 integrases. Proc Natl Acad Sci U S A. 104:3312–3317. 10.1073/pnas.0611511104. 17360644 PMC1805588

[jkag077-B5] Brown JL, Price JD, Erokhin M, Kassis JA. 2023. Context-dependent role of Pho binding sites in Polycomb complex recruitment in Drosophila. Genetics. 224:iyad096. 10.1093/genetics/iyad096. 37216193 PMC10411561

[jkag077-B6] Chung HL et al 2022. De novo variants in EMC1 lead to neurodevelopmental delay, cerebellar degeneration and affect glial function in Drosophila. Hum Mol Genet. 31:3231–3244. 10.1093/hmg/ddac053. 35234901 PMC9523557

[jkag077-B7] Chung HL et al 2020. Loss- or gain-of-function mutations in ACOX1 cause axonal loss via different mechanisms. Neuron. 106:589–606.e6. 10.1016/j.neuron.2020.02.021. 32169171 PMC7289150

[jkag077-B8] Cook K . 2025. The underappreciated, underrecognized problem of fourth chromosome trisomy in Drosophila melanogaster stocks and a simple, general method for building diplo-4 stocks from triplo-4 stocks. MicroPubl Biol. 10.17912/micropub.biology.001606. 10.17912/micropub.biology.001606. PMC12082342 40385372

[jkag077-B9] Diao F et al 2015. Plug-and-play genetic access to Drosophila cell types using exchangeable exon cassettes. Cell Rep. 10:1410–1421. 10.1016/j.celrep.2015.01.059. 25732830 PMC4373654

[jkag077-B10] Diao F, White BH. 2012. A novel approach for directing transgene expression in Drosophila: T2A-Gal4 in-frame fusion. Genetics. 190:1139–1144. 10.1534/genetics.111.136291. 22209908 PMC3296248

[jkag077-B11] Entchev EV, Schwabedissen A, González-Gáitan M. 2000. Gradient formation of the TGF-beta homolog Dpp. Cell. 103:981–992. 10.1016/S0092-8674(00)00200-2. 11136982

[jkag077-B421] Estella C, Voutev R, Mann RS. 2012. A dynamic network of morphogens and transcription factors patterns the fly leg. Curr Top Dev Biol. 98:173–198. 10.1016/j.devcel.2009.06.008. 22305163 PMC3918458

[jkag077-B12] Fuentes-Medel Y et al 2012. Integration of a retrograde signal during synapse formation by glia-secreted TGF-β ligand. Curr Biol. 22:1831–1838. 10.1016/j.cub.2012.07.063. 22959350 PMC3605899

[jkag077-B13] Gabriele M et al 2017. YY1 haploinsufficiency causes an intellectual disability syndrome featuring transcriptional and chromatin dysfunction. Am J Hum Gen. 100:907–925. 10.1016/j.ajhg.2017.05.006. PMC5473733 28575647

[jkag077-B14] Gibson DG et al 2009. Enzymatic assembly of DNA molecules up to several hundred kilobases. Nat Methods. 6:343–345. 10.1038/nmeth.1318. 19363495

[jkag077-B15] Goldsmith SL et al 2022. New resources for the Drosophila fourth chromosome: FRT101F enabled mitotic clones and *Bloom syndrome helicase* enabled meiotic recombination. G3 (Bethesda). 12:jkac019. 10.1093/g3journal/jkac019. 35084488 PMC8982423

[jkag077-B16] Gratz S et al 2013. Genome engineering of Drosophila with the CRISPR RNA-guided Cas9 nuclease. Genetics. 194:1029–1035. 10.1534/genetics.113.152710. 23709638 PMC3730909

[jkag077-B17] Harsh S, Liu HY, Bhaskar PK, Rushlow C, Bach EA. 2025. Post-transcriptional suppression of the pioneer factor Zelda protects the adult Drosophila testis from activation of the ovary program. PLoS Biol. 23:e3003535. 10.1371/journal.pbio.3003535. 41411219 PMC12714197

[jkag077-B18] Holguera I, Desplan C. 2018. Neuronal specification in space and time. Science. 362:176–180. 10.1126/science.aas9435. 30309944 PMC6368964

[jkag077-B19] Hoyer N et al 2018. Ret and substrate-derived TGF-beta Maverick regulate space-filling dendrite growth in Drosophila sensory neurons. Cell Rep. 24:2261–2272.e5. 10.1016/j.celrep.2018.07.092. 30157422 PMC6191840

[jkag077-B20] Iovino N, Pane A, Gaul U. 2009. miR-184 has multiple roles in Drosophila female germline development. Dev Cell. 17:123–133. 10.1016/j.devcel.2009.06.008. 19619497

[jkag077-B21] Kumar JP . 2009. The molecular circuitry governing retinal determination. Biochim Biophys Acta. 1789:306–314. 10.1016/j.bbagrm.2008.10.001. 19013263 PMC2700058

[jkag077-B420] Kropp PA, Gannon M. 2016. Onecut transcription factors in development and disease. Trends Dev Biol. 9:43–57. 28018056 PMC5176019

[jkag077-B22] Lee P et al 2018. A gene-specific T2A-GAL4 library for Drosophila. eLife. 7:e35574. 10.7554/eLife.35574. 29565247 PMC5898912

[jkag077-B23] Lee T, Luo L. 1999. Mosaic analysis with a repressible cell marker for studies of gene function in neuronal morphogenesis. Neuron. 22:451–461. 10.1016/S0896-6273(00)80701-1. 10197526

[jkag077-B24] Li X et al 2013. Temporal patterning of Drosophila medulla neuroblasts controls neural fates. Nature. 498:456–462. 10.1038/nature12319. 23783517 PMC3701960

[jkag077-B25] Li-Kroeger D et al 2018. An expanded toolkit for gene tagging based on MiMIC and scarless CRISPR tags in Drosophila. eLife. 7:e38709. 10.7554/eLife.38709. 30091705 PMC6095692

[jkag077-B26] Liu Z, Ambros V. 1989. Heterochronic genes control the stage-specific initiation and expression of the Dauer larva program in *Caenorhabditis elegans*. Genes Dev. 3:2039–2049. 10.1101/gad.3.12b.2039. 2628162

[jkag077-B27] Myers L, Perera H, Alvarado MG, Kidd T. 2018. The Drosophila Ret gene functions in the stomatogastric nervous system with the Maverick TGF-β ligand and the Gfrl co-receptor. Development. 145:dev157446. 10.1242/dev.157446. 29361562 PMC5818002

[jkag077-B28] Nagarkar-Jaiswal S et al 2015. A library of MiMICs allows tagging of genes and reversible, spatial and temporal knockdown of proteins in Drosophila. eLife. 4:e05338. 10.7554/eLife.05338. 25824290 PMC4379497

[jkag077-B29] Nguyen D, Rohrbaugh M, Lai ZC. 2000. The Drosophila homolog of Onecut homeodomain proteins is a neural-specific transcriptional activator with a potential role in regulating neural differentiation. Mech Dev. 97:57–72. 10.1016/S0925-4773(00)00431-7. 11025207

[jkag077-B30] Okamoto N, Nishimori Y, Nishimura T. 2012. Conserved role for the Dachshund protein with Eyeless in insulin expression. Proc Natl Acad Sci U S A. 109:2406–2411. 10.1073/pnas.1116050109. 22308399 PMC3289324

[jkag077-B31] Öztürk-Çolak A et al 2024. FlyBase: updates to the Drosophila genes and genomes database. Genetics. 227:. 10.1093/genetics/iyad211. PMC11075543 38301657

[jkag077-B32] Pan L, Broadie KS. 2007. Drosophila fragile X protein and mGluR convergently regulate the synaptic ratio of ionotropic glutamate receptor subclasses. J Neurosci. 27:12378–12389. 10.1523/JNEUROSCI.2970-07.2007. 17989302 PMC6673270

[jkag077-B414] Perkins LA et al 2015. The transgenic RNAi project at Harvard Medical School: resources and validation. Genetics. 201:843–852. 26320097 10.1534/genetics.115.180208 PMC4649654

[jkag077-B33] Protasova MS, Andreeva TV, Klyushnikov SA, Illarioshkin SN, Rogaev EI. 2023. Genetic variant in GRM1 underlies congenital cerebellar ataxia with no obvious intellectual disability. Int J Mol Sci. 24:1551. 10.3390/ijms24021551. 36675067 PMC9865416

[jkag077-B34] Punzo C et al 2004. Functional divergence between eyeless and toy in *Drosophila melanogaster*. Development. 131:3943–3953. 10.1242/dev.01278. 15253940

[jkag077-B35] Quasney C, Griffith LC. 2025. Degradation of CaMKII is stimulated by its active conformation. J Biol Chem. 301:110601. 10.1016/j.jbc.2025.110601. 40818612 PMC12624777

[jkag077-B36] Raja KKB, Yeung K, Li Y, Chen R, Mardon G. 2024. A single cell RNA sequence atlas of the early Drosophila larval eye. BMC Genomics. 25:616. 10.1186/s12864-024-10423-x. 38890587 PMC11186242

[jkag077-B37] Sapkota D et al 2014. Onecut1 and Onecut2 regulate early retinal cell fates during development. Proc Natl Acad Sci U S A. 111:E4086–E4095. 10.1073/pnas.1405354111. 25228773 PMC4191802

[jkag077-B38] Sipe C, Siegrist S. 2017. Eyeless uncouples mushroom body neuroblast proliferation from dietary amino acids in Drosophila. eLife. 6:e26343. 10.7554/eLife.26343. 28826476 PMC5576483

[jkag077-B39] Spratford C, Kumar J. 2014. Hedgehog and extramacrochaetae in the Drosophila eye: an irresistible force meets an immovable object. Fly (Austin). 8:36–42. 10.4161/fly.27691. 24406336 PMC3974893

[jkag077-B40] Stinchfield MJ, Gadagkar SR, O’Connor MB, Newfeld SJ. 2026. Both isoforms of Drosophila ApoLpp (ApoB) cross the blood-brain barrier in adults. Genetics. 232:iyaf224. 10.1093/genetics/iyaf224. 41099152 PMC12774823

[jkag077-B41] Stinchfield MJ et al 2024. Fourth chromosome resource project: a comprehensive resource for genetic analysis in Drosophila that includes humanized stocks. Genetics. 226:iyad201. 10.1093/genetics/iyad201. 37981656 PMC10847715

[jkag077-B42] Sun F et al 2004. cis-acting determinants of heterochromatin formation on Drosophila chromosome 4. Mol Cell Biol. 24:8210–8220. 10.1128/MCB.24.18.8210-8220.2004. 15340080 PMC515050

[jkag077-B43] Tan Y, Yu D, Busto GU, Wilson C, Davis RL. 2013. Wnt signaling is required for long-term memory formation. Cell Rep. 4:1082–1089. 10.1016/j.celrep.2013.08.007. 24035392 PMC4083693

[jkag077-B44] Tran N et al 2018. CORL expression and function in insulin producing neurons reversibly influences adult longevity in Drosophila. G3 (Bethesda). 8:2979–2990. 10.1534/g3.118.200572. 30006413 PMC6118311

[jkag077-B45] van Tienen LM, Mieszczanek J, Fiedler M, Rutherford TJ, Bienz M. 2017. Constitutive scaffolding of multiple Wnt enhanceosome components by Legless/BCL9. Elife. 6:e20882. 10.7554/eLife.20882. 28296634 PMC5352222

[jkag077-B46] Venken KJT, He Y, Hoskins R, Bellen HJ. 2006. P[acman]: a BAC transgenic platform for targeted insertion of large DNA fragments in *D. melanogaster*. Science. 314:1747–1751. 10.1126/science.1134426. 17138868

[jkag077-B422] Verheul TCJ, van Hijfte L, Perenthaler E, Barakat TS. 2020. The why of YY1: mechanisms of transcriptional regulation by Yin Yang 1. Front Cell Dev Biol. 8:592164. 33102493 10.3389/fcell.2020.592164 PMC7554316

[jkag077-B47] Weasner BM et al 2025. A new *Drosophila melanogaster* research resource: CRISPR-induced mutations for clonal analysis of fourth chromosome genes. G3 (Bethesda). 15:jkaf006. 10.1093/g3journal/jkaf006. 39804955 PMC11917476

[jkag077-B48] Weasner BM, Weasner BP, Neuman SD, Bashirullah A, Kumar JP. 2016. Retinal expression of the Drosophila eyes absent gene is controlled by several cooperatively acting cis-regulatory elements. PLoS Genet. 12:e1006462. 10.1371/journal.pgen.1006462. 27930646 PMC5145141

[jkag077-B49] Wisotzkey RG, Newfeld SJ. 2020. TGF-β prodomain alignments reveal unexpected cysteine conservation consistent with phylogenetic predictions of cross-subfamily heterodimerization. Genetics. 214:447–465. 10.1534/genetics.119.302255. 31843757 PMC7017013

[jkag077-B50] Yamamoto S et al 2014. A Drosophila genetic resource study mechanisms underlying human genetic diseases. Cell. 159:200–214. 10.1016/j.cell.2014.09.002. 25259927 PMC4298142

[jkag077-B51] Yamamoto S, Kanca O, Wangler MF, Bellen HJ. 2024. Integrating non-mammalian model organisms in the diagnosis of rare genetic diseases in humans. Nat Rev Genet. 25:46–60. 10.1038/s41576-023-00633-6. 37491400

[jkag077-B52] Yang T, Cheng L, Kain SR. 1996. Optimized codon usage and chromophore mutations provide enhanced sensitivity with the green fluorescent protein. Nucleic Acids Res. 24:4592–4593. 10.1093/nar/24.22.4592. 8948654 PMC146266

[jkag077-B53] Zhang X, Koolhaas WH, Schnorrer F. 2014. A versatile two-step CRISPR- and RMCE-based strategy for efficient genome engineering in Drosophila. G3 (Bethesda). 4:2409–2418. 10.1534/g3.114.013979. 25324299 PMC4267936

[jkag077-B54] Zhu J, Palliyil S, Ran C, Kumar JP. 2017. Drosophila Pax6 promotes development of the entire eye-antennal disc, thereby ensuring proper adult head formation. Proc Natl Acad Sci U S A. 114:5846–5853. 10.1073/pnas.1610614114. 28584125 PMC5468661

