# Supplementary material for: Genetic analyses enabled by the fourth chromosome resource project reveal unexpected mutant phenotypes and suggest new disease models
Source: G3 (Bethesda). 2026 Mar 28;16(6):jkag077. doi: 10.1093/g3journal/jkag077 (PMC13232516; doi:10.1093/g3journal/jkag077)
Supplement: jkag077_Supplementary_Data [file jkag077_supplementary_data.pdf]

Supplemental Information Six Figures and Seven Tables

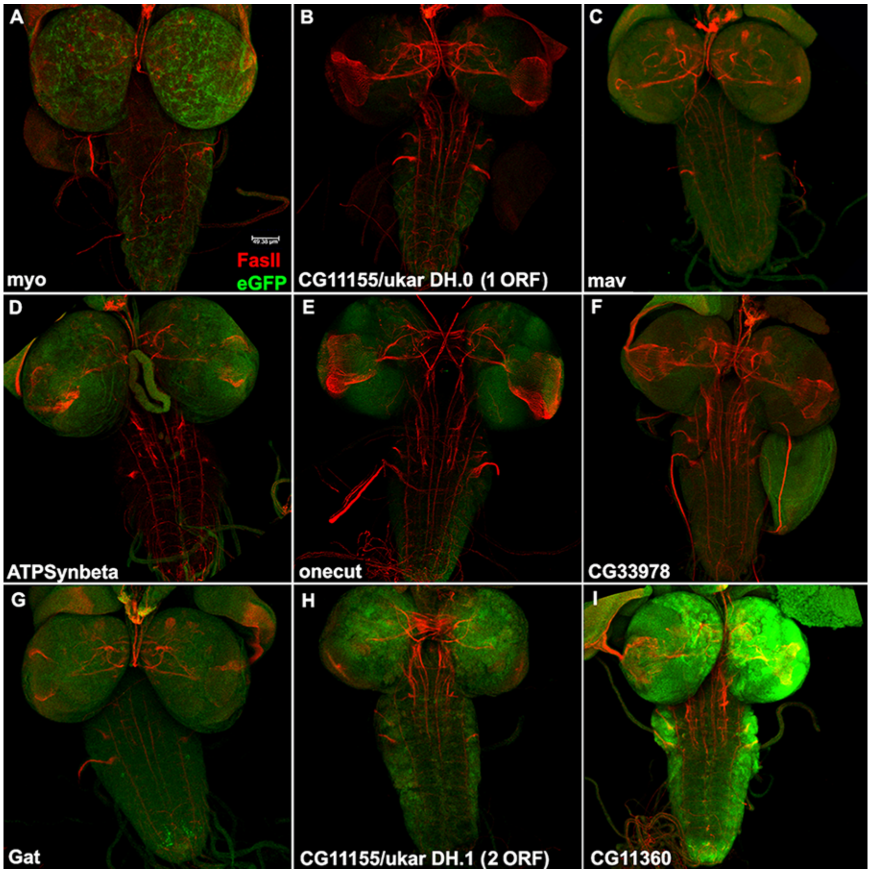

Fig. S1. New DoubleHeader converted eGFP protein trap images. Third instar larval brain, ventral cord, associated glands and imaginal discs reflecting eGFP and FasII (n = 3 per genotype). A scale bar is shown. Antibody detection of eGFP from a converted CRIMIC. Movies of each confocal image are available from FCRP. A) Myoglianin with moderate restricted expression in brain and ventral cord. B) CG11155/Ukar reading frame zero - weak restricted expression in brain and ventral cord. Inserted in intron 11, the converted CR01527 is predicted to trap one of three open reading frames (Ukar-PB). The other reading frame in phase one is shown below in H. C) Maverick with diffuse expression in brain plus a few spots of stronger expression in the ventral cord. D) ATPSynbeta with moderate expression in brain. E) Onecut with weak expression in brain and ventral cord. F) CG33978 with weak expression in brain and ventral cord plus moderate expression in wing and eye discs. G) Gat with weak expression in brain and ventral cord plus a small area of strong expression in the ring gland. H) CG11155/Ukar reading frame one - moderate expression in brain and ventral cord. Inserted in intron three, the converted CR70473 is predicted to trap two open reading frames (Ukar-PA, Ukar-PD); the other frame in phase zero is shown above in B. I) CG11360 with strong eGFP expression in brain, ventral cord and eye discs.

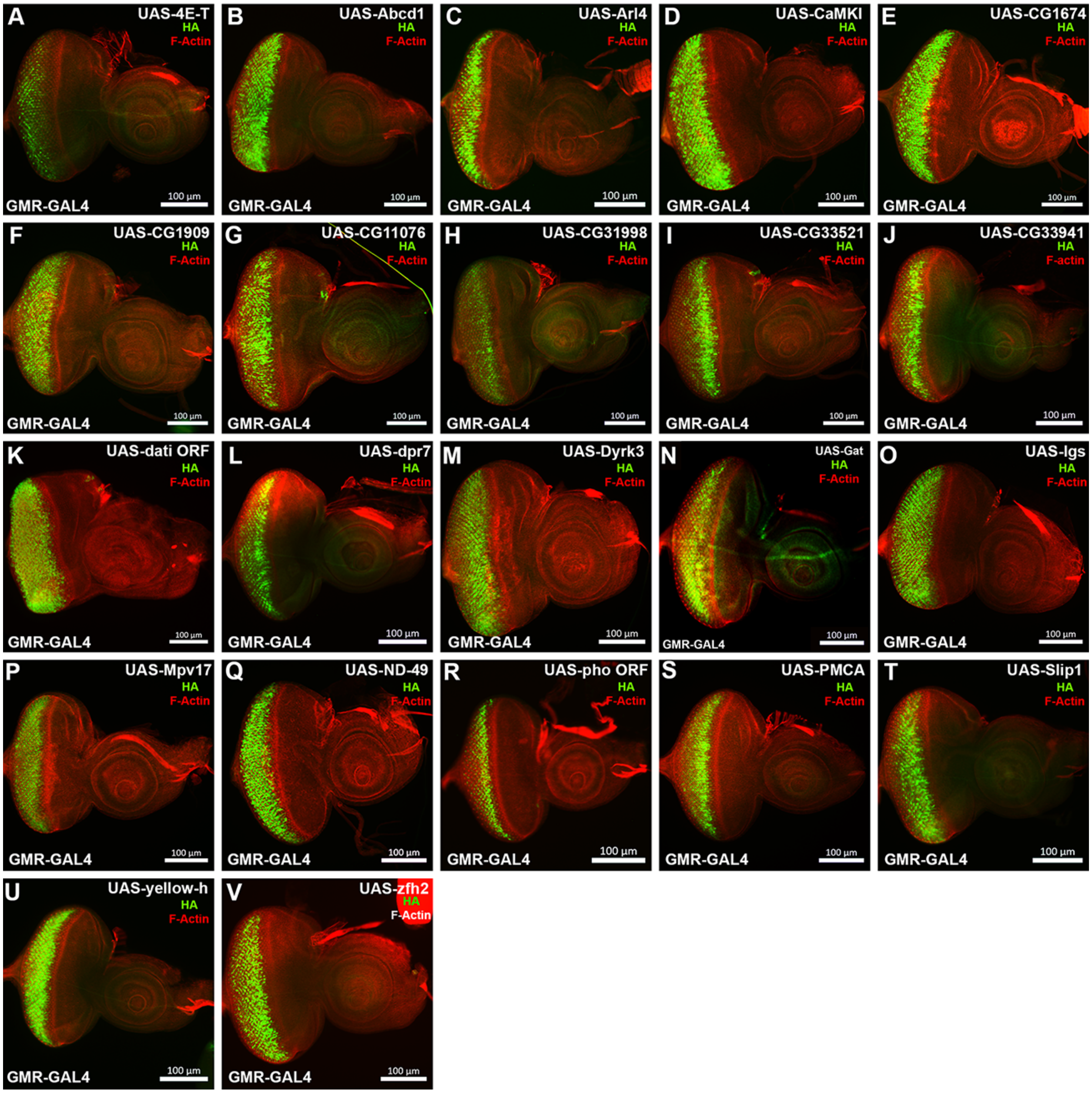

Fig. S2. Representative 3'-HA tag verification images for UAS.fly cDNA stocks. A-V) Third instar eye-antennal discs displaying HA (green) to detect a tagged fly cDNA and phalloidin (red) to mark F-Actin (n=3 per genotype). Anterior to the right with a scale bar present. GMR.GAL4 (BL8605) driving the indicated cDNA. 3'-HA expression is visible in all. An update with new UAS.fly cDNA stocks is in **Table S3A**. A summary of HA expression data for all tagged fly cDNA stocks is in **Table S4A**. Note if a fly protein is known to be sensitive to a carboxy-terminal tag, such as Activin-β, then none was added.

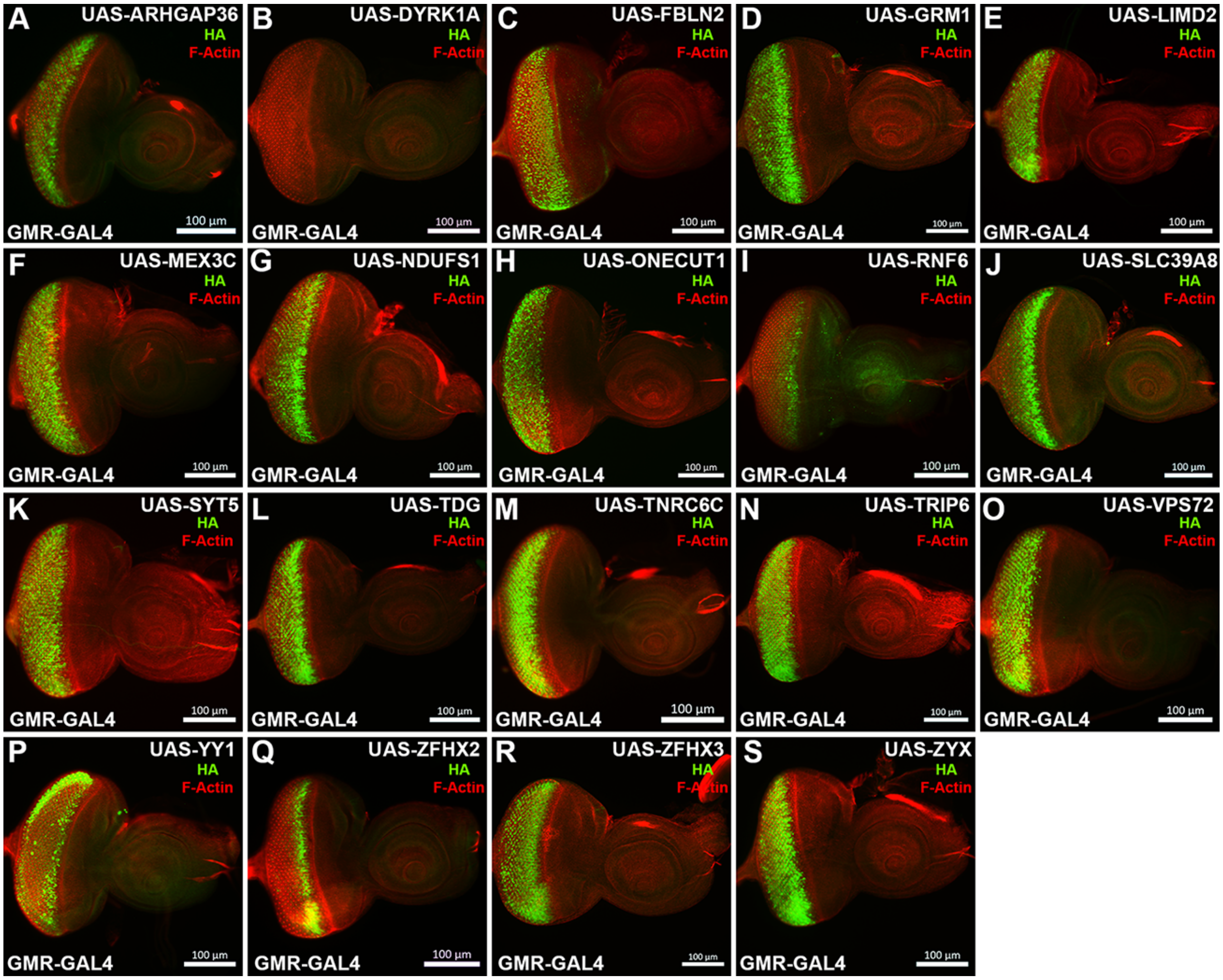

Fig. S3. Representative 3'-HA tag verification images for UAS.human cDNA stocks. A-S) Third instar eye-antennal discs as in **Fig. S2**. 3'-HA expression is visible in all except discs except DYRK1A (B; replacement transgene under construction). An update with new UAS.human cDNA stocks is in **Table S3B**. A summary of HA expression data for all tagged human cDNA stocks in Bloomington is in **Table S4B**. Note if a human protein is known to be sensitive to a carboxy-terminal tag, such as TGFB1, then none was added.

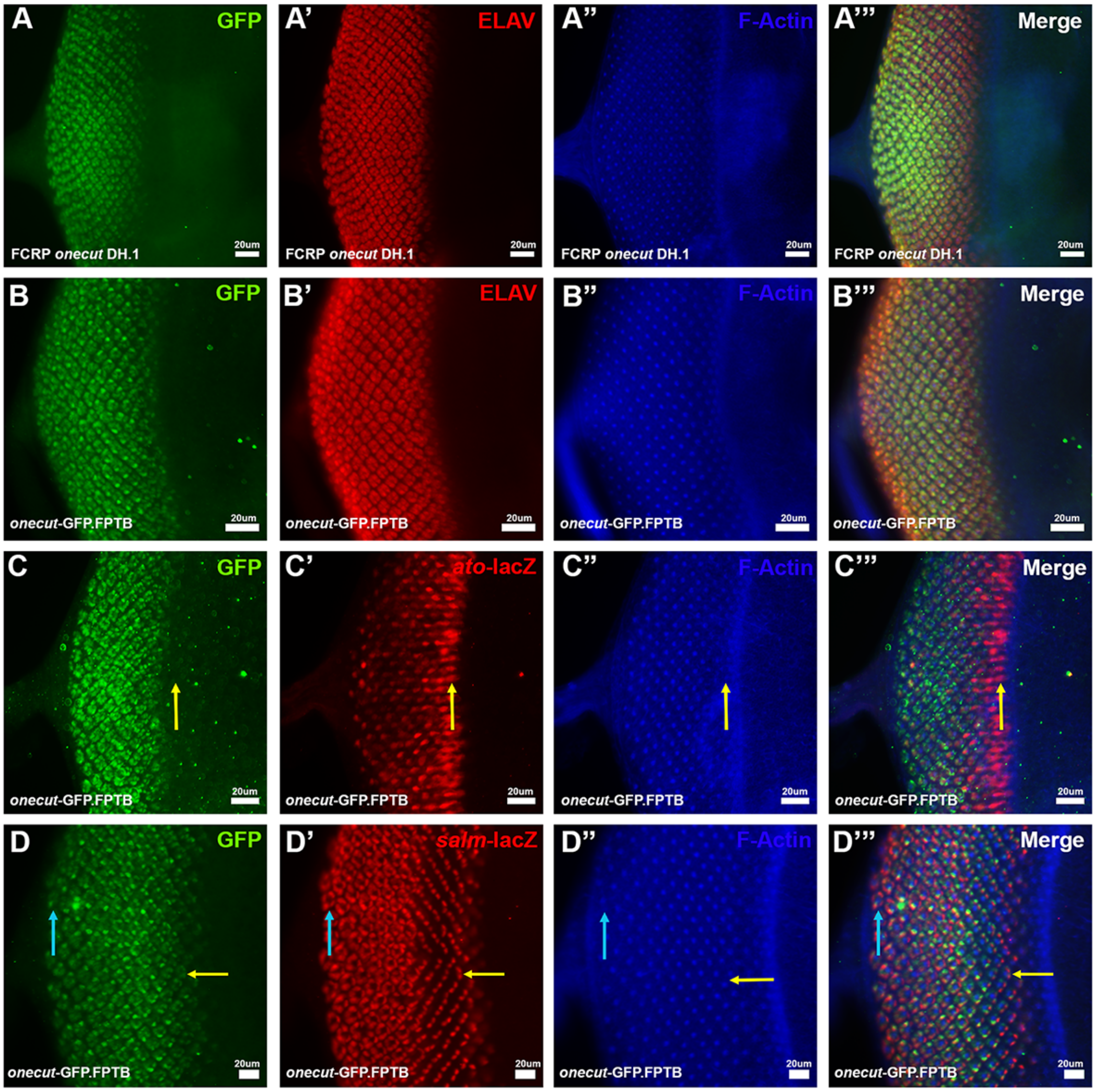

Fig. S4. Fly Onecut eye disc expression begins between R8 and R3/R4 specification. A-D) High magnification view of the differentiated region of third instar eye-antennal discs. A-A"') Onecut eGFP (green) created by FCRP. B-B"', C-C", D-D"') Genomic BAC containing GFP tagged Onecut (green; BL83670). A',B') Elav in all photoreceptors. C') *ato*-lacZ marks the R8 photoreceptor (yellow arrow in C-C"'). D') *salm*-lacZ marks the R3 and R4 photoreceptors (yellow arrow in D-D"') and the most posterior cone cells (blue arrow in D-D"'). A"-D") Individual ommatidial clusters display F-Actin. C"'-D"') Merged view shows Onecut in all photoreceptors after the initial onset of *ato-lacZ* and coincident with the onset of Elav and *salm*-lacZ. D'") Onecut is also present in the most posterior cone cells (blue arrow).

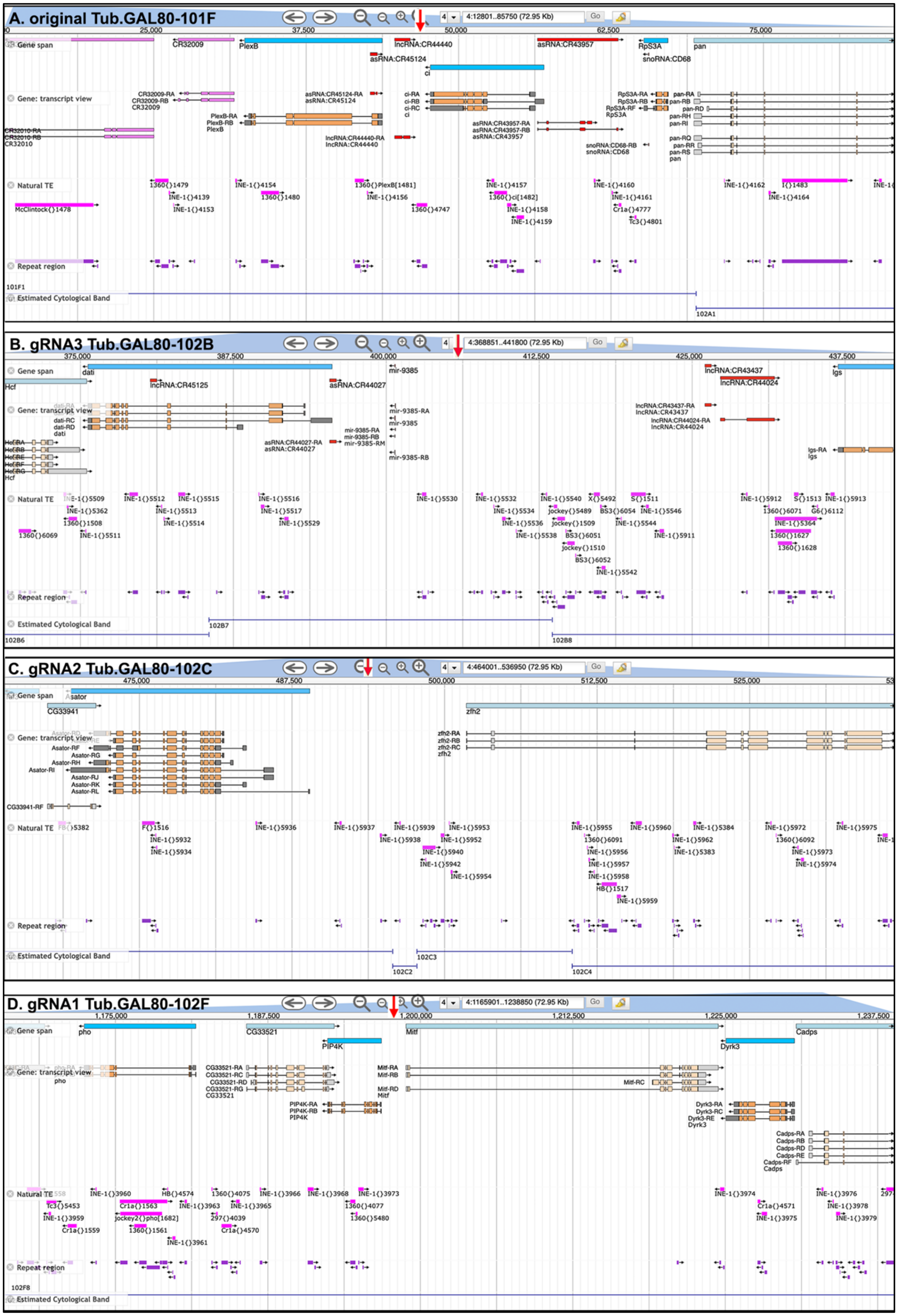
A. original Tub.GAL80-101F
B. gRNA3 Tub.GAL80-102B
C. gRNA2 Tub.GAL80-102C
D. gRNA1 Tub.GAL80-102F

Fig. S5. Insertion site details for the original and three new Tub.GAL80 transgenes on the FRT101F chromosome. Images were downloaded from Flybase J-Browse and display the fourth chromosome at four locations containing a CRISPR inserted Tub.GAL80 transgene. Each number line reflects base pair numbering with the centromere to the left. The initial and final base pair of each 72.95kb region are in the box above the number line. Base pair numbering and all annotations from release 6.54 (12/12/2023) with gene span, gene transcript view, natural transposable elements, repeat region and estimated cytological band shown. A) As reported in Goldsmith et al. (2022), the original Tub.GAL80-101F immediately distal to FRT101F at 46,995 base pairs between *PlexB* and *ci* was inserted into a 1360 transposable element. B) New CRISPR insertion of Tub.GAL80-102B at 405,575 base pairs is between *dati* and *lgs* in an unpopulated region. C) New CRISPR insertion of Tub.GAL80-102C at 489,878 base pairs is between *Asator* and *zfh2* in an unpopulated region. C) New CRISPR insertion of Tub.GAL80-102F at 1,197,868 base pairs is between *PIP4K* and *Mitf* in an unpopulated region.

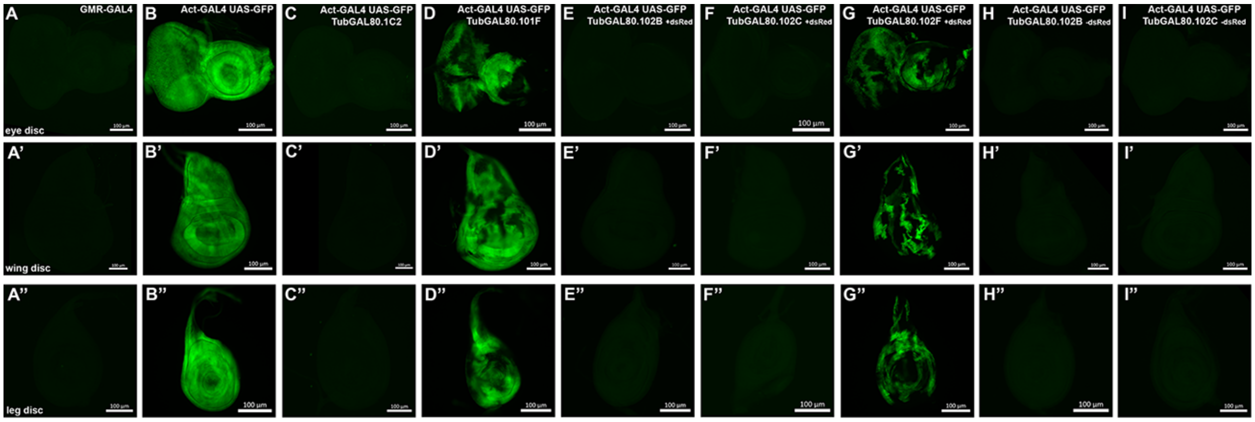

Fig. S6. FRT101F Tub.GAL80-102B and -102C fully suppress Actin5C.GAL4 driven UAS.GFP in imaginal discs. Third instar eye (top row), wing (middle row) and leg discs (bottom row) are shown. Each column is a distinct genotype. A-A") GMR.GAL4 alone reveals a very low background level of autofluorescence. B-B") Actin5C.GAL4 driving UAS.GFP ubiquitously is the negative control for suppression. C-C") Actin5C.GAL4 driving UAS.GFP ubiquitously in the presence of Tub.GAL80 on the X chromosome (Addgene plasmid 17748; tubulin 1alpha promoter linked to the GAL80 coding region; Lee & Luo 1999) is the positive control for suppression. D-D") Original FRT101F Tub.GAL80-101F developed variegation in suppression of Actin5C.GAL4 driving UAS.GFP. E-E") New FRT101F Tub.GAL80-102B fully suppresses Actin5C.GAL4 driving UAS.GFP. F-F") New FRT101F Tub.GAL80-102C fully suppresses Actin5C.GAL4 driving UAS.GFP. G-G") New FRT101F Tub.GAL80-102F variegates in suppression of Actin5C.GAL4 driving UAS.GFP. H-H") New FRT101F.Tub.GAL80-102B with DsRed removed fully suppresses Actin5C.GAL4 driving UAS.GFP. I-I") New FRT101F.Tub.GAL80-102C with DsRed removed fully suppresses Actin5C.GAL4 driving UAS.GFP.

**Table S1. Glossary of genomic technology with citations for further information**
**MiMIC transgene**: A Minos-mediated integration cassette. This transgene contains a Minos inverted repeat, attP site, Splice Acceptor, stop codons in all three reading frames, eGFP coding sequence, poly A, Splice Donor, attP site, yellow transformation marker and Minos inverted repeat (Bellen et al. 2011). This transgene is amenable to RMCE (defined below). Minos is a transposable element from the fruit fly *Drosophila hydei* (Pavlopoulos et al. 2007).
**CRIMIC transgene**: Cas9 homology directed repair (CRISPR) mediated integration cassette. This transgene contains an attP site, FRT site, Splice Acceptor, T2A peptide (defined below), GAL4 coding region, polyA tail, Splice Donor, FRT site, attP site and the 3XP3-eGFP transformation marker (Li-Kroeger et al. 2018). This transgene is amenable to RMCE.
**RMCE method:** Recombination Mediated Cassette Exchange. Developed for a T2A.GAL4 (defined below) cassette carried on injected donor plasmids that are swapped for the yellow transformation marker of MiMIC trangenes in coding region introns (Diao et al. 2015). Subsequently the method was adapted to a simple crossing scheme. This was done by creating stocks with each of the three frames of DoubleHeader donor plasmids plus maternal sources of Cre recombinase to release them and ϕC31 integrase to complete the swap. The method targets MiMIC yellow or CRIMIC 3xP3-eGFP transformation markers (Li-Kroeger et al. 2018).
**DoubleHeader transgene**: A donor plasmid (or transgene) that contains a loxP site, attB site, Splice Acceptor, five protein tags (EGFP-FIAsH-StrepII-TEV-3XFlag), and a Splice Donor in one orientation. The other orientation has a Splice Acceptor, T2A peptide (defined below), GAL4 coding region, poly A tail and the same Splice Donor (Li-Kroeger et al 2018).
**T2A.GAL4 stock:** Gene trap stock that has an artificial exon from a DoubleHeader plasmid or transgene donor matching the reading frame of the endogenous protein in a coding region exon. The exon contains a Splice Acceptor, 2A peptide from the insect virus *Thosea asigna* that causes ribosomal skipping, GAL4 coding region, and a Splice Donor (Diao and White 2012). Formal names for these stocks include the T2A.GAL4 abbreviation TG4 and the DoubleHeader reading frame (0, 1 or 2) . For example, *mav* T2A.Gal4 is TI{CRIMIC.TG4.0}mavCR70166-TG4.0.
**eGFP stock:** Protein trap stock that has an artificial exon from a DoubleHeader plasmid or transgene donor matching the reading frame of the endogenous protein in a coding region exon. The exon contains a Splice Acceptor, five protein tags (EGFP-FIAsH-StrepII-TEV-3XFlag, and a Splice Donor (Nagarkar-Jaiswal et al. 2015). Formal names for these stocks include the five tag abbreviation GFSTF and the DoubleHeader reading frame. For example, *mav* eGFP is TI{DH.0}mavCR70166-DH.PT-GFSTF.0.
**3xP3 CRIMIC RMCE target:** An eye-specific artificial promoter containing three copies of the P3 binding site for Eyeless from the promoter of *rhodopsin1* employed as a transformation marker (Horn and Wimmer 2000).
**MARCM method:** Mosaic Analysis with a Repressible Cell Marker. This is a recent innovation in a long-standing effort to create a single mutant cell in an otherwise wild type individual. The goal is to facilitate single cell analysis of the mutant phenotype in the original cell and its descendants called clones (the general method is named clonal analysis). All methods for clonal analysis require a heterozygous mutant genotype. This specific method combines: 1) GAL4-UAS system to drive the ubiquitous expression of both GAL4 and the GAL4 repressor Gal80 and 2) FLP-FRT activation of mitotic recombination to homozygose the GAL80 repressor in one daughter cell and the mutation in the other. In the daughter cell that is homozygous mutant, GAL4 is no longer repressed and can mark that cell via UAS.GFP (Lee and Luo 1999).

Table S2

FCRP: Update to DoubleHeader converted CRIMICs with Bloomington & Kyoto stock number plus image webpage			
Bloom	Kyoto	Genotype	Flypush larval brain expression
97733	118953	y[1] w[1118]; TI{DH.2}sv[CR00370-DH.PTGFSTF.2]/ In(4)ci[D], ci[D] pan[ciD]	https://flypush.research.bcm.edu/pscreen/rmce/rmce.php?entry=RM20024
97734	118954	y[1] w[1118]; TI{DH.1}CG31999[CR01673-DH.PT-GFSTF.1]	https://flypush.research.bcm.edu/pscreen/rmce/rmce.php?entry=RM20025
97735	118955	y[1] w[1118]; TI{DH.1}Rad23[CR01823-DH.PT-GFSTF.1]	https://flypush.research.bcm.edu/pscreen/rmce/rmce.php?entry=RM20026
97736	118956	y[1] w[1118]; TI{DH.2}CG31998[CR01849-DH.PT-GFSTF.2]	https://flypush.research.bcm.edu/pscreen/rmce/rmce.php?entry=RM20027
97737	118957	y[1] w[1118]; TI{DH.2}CG32017[CR01850-DH.PT-GFSTF.2]	https://flypush.research.bcm.edu/pscreen/rmce/rmce.php?entry=RM20028
97738	118958	y[1] w[1118]; TI{DH.0}Ank[CR01879-DH.PT-GFSTF.0]	https://flypush.research.bcm.edu/pscreen/rmce/rmce.php?entry=RM20029
97739	118959	y[1] w[1118]; TI{DH.1}ey[CR02257-DH.PT-GFSTF.1]	https://flypush.research.bcm.edu/pscreen/rmce/rmce.php?entry=RM20030
97740	118960	y[1] w[1118]; TI{DH.0}Arl4[CR02574-DH.PT-GFSTF.0]	https://flypush.research.bcm.edu/pscreen/rmce/rmce.php?entry=RM20031
97741	118961	y[1] w[1118]; TI{DH.1}CaMKI[CR02670-DH.PT-GFSTF.1]	https://flypush.research.bcm.edu/pscreen/rmce/rmce.php?entry=RM20032
97742	118962	y[1] w[1118]; TI{DH.0}Slip1[CR02779-DH.PT-GFSTF.0]	https://flypush.research.bcm.edu/pscreen/rmce/rmce.php?entry=RM20033
97743	118963	y[1] w[1118]; TI{DH.1}anne[CR70036-DH.PT-GFSTF.1]	https://flypush.research.bcm.edu/pscreen/rmce/rmce.php?entry=RM20034
97744	118964	y[1] w[1118]; TI{DH.1}apolpp[CR70471-DH.PT-GFSTF.1]/ In(4)ci[D], ci[D] pan[ciD]	https://flypush.research.bcm.edu/pscreen/rmce/rmce.php?entry=RM20035
97745	118965	y[1] w[1118]; TI{DH.1}CG31997[CR70475-DH.PT-GFSTF.1]	https://flypush.research.bcm.edu/pscreen/rmce/rmce.php?entry=RM20036
97746	118966	y[1] w[1118]; TI{DH.0}CG33941[CR70477-DH.PT-GFSTF.0]	https://flypush.research.bcm.edu/pscreen/rmce/rmce.php?entry=RM20037
97747	118967	y[1] w[1118]; TI{DH.0}gw[CR70482-DH.PT-GFSTF.0]/ TI{GMR-HMS04515}Gat[eya]	https://flypush.research.bcm.edu/pscreen/rmce/rmce.php?entry=RM20038
97748	118968	y[1] w[1118]; TI{DH.0}JYalpha[CR70483-DH.PT-GFSTF.0]/ In(4)ci[D], ci[D] pan[ciD]	https://flypush.research.bcm.edu/pscreen/rmce/rmce.php?entry=RM20039
97749	118969	y[1] w[1118]; TI{DH.0}PIP4K[CR70484-DH.PT-GFSTF.0]/ In(4)ci[D], ci[D] pan[ciD]	https://flypush.research.bcm.edu/pscreen/rmce/rmce.php?entry=RM20040
97750	118970	y[1] w[1118]; TI{DH.1}PlexA[CR70485-DH.PT-GFSTF.1]/ TI{GMR-HMS04515}Gat[eya]	https://flypush.research.bcm.edu/pscreen/rmce/rmce.php?entry=RM20041
97751	118971	y[1] w[1118]; TI{DH.2}zfh2[CR70564-DH.PT-GFSTF.2]/ TI{GMR-HMS04515}Gat[eya]	https://flypush.research.bcm.edu/pscreen/rmce/rmce.php?entry=RM20042
97752	118972	y[1] w[1118]; TI{DH.1}Gyf[CR70565-DH.PT-GFSTF.1]/ In(4)ci[D], ci[D] pan[ciD]	https://flypush.research.bcm.edu/pscreen/rmce/rmce.php?entry=RM20043
97753	118973	y[1] w[1118]; TI{DH.1}4E-T[CR92248-DH.PT-GFSTF.1]	https://flypush.research.bcm.edu/pscreen/rmce/rmce.php?entry=RM20044
97754	118974	y[1] w[1118]; TI{DH.1}lgs[CR92249-DH.PT-GFSTF.1]	https://flypush.research.bcm.edu/pscreen/rmce/rmce.php?entry=RM20045
97755	118975	y[1] w[1118]; Mi{DH.0}fuss[MI03207-DH.PT-GFSTF.0]	https://flypush.research.bcm.edu/pscreen/rmce/rmce.php?entry=RM20046
97756	118976	y[1] w[1118]; Mi{DH.1}CaMKII[MI03976-DH.GT-TG4.1]/ TI{GMR-HMS04515}Gat[eya]	https://flypush.research.bcm.edu/pscreen/rmce/rmce.php?entry=RM20047

97756	118977	y[1] w[1118]; Mi{DH.1}CaMKII[MI03976-DH.GT-TG4.1]/ TI{GMR-HMS04515}Gat[eya]	https://flypush.research.bcm.edu/pscreen/rmce/rmce.php?entry=RM20047
			**Notes for brain images in Fig. S1**
600215	118960	y[1] w[1118]; TI{DH.1}myo[CR02262-DH.PT-GFSTF.1]/ TI{GMR-HMS04515}Gat[eya]	Kyoto 118960 fourth balancer is ciD; these males are gas sensitive
603168	119136	y[1] w[1118]; Ti{DH.0}CG11155/ukar[CR01527-DH.PT-GFSTF.0]	Due to alternative splicing - reading frame zero captures one annotated ORF (PB)
603169	119137	y[1] w[1118]; Ti{DH.0}mav[CR70166-DH.PT-GFSTF.0]	
603170	119150	y[1] w[1118]; Ti{DH.1}ATPsynbeta[CR02261-DH.PT-GFSTF.1]/ TI{GMR-HMS04515}Gat[eya]	
603171	119151	y[1] w[1118]; Ti{DH.0}Gat[CR01191-DH.PT-GFSTF.0]	
603172	119152	y[1] w[1118]; Ti{DH.1}CG33978[CR70478-DH.PT-GFSTF.1]/ TI{GMR-HMS04515}Gat[eya]	
603173	119153	y[1] w[1118]; Ti{DH.1}onecut[CR70949-DH.PT-GFSTF.1]	
605863	119310	y[1] w[1118]; TI{DH.1}CG11155/ukar[CR70473-DH.PT-GFSTF.1]	Due to alternative splicing - reading frame one captures two annotated ORFs (PA,PD)
605864	119311	y[1] w[1118]; TI{DH.1}CG11360[CR02793-DH.PT-GFSTF.1]	

Table S3

FCRP: Update to UAS.fly cDNA & UAS.human cDNA stocks with Bloomington & Kyoto stock numbers		
**3A: UAS.fly cDNA**		
**Bloomington**	**Kyoto**	**Genotype**
99865	119075	y[1] w[1118]; PBac{y[+mDint2] w[+mC]=UAS-yellow-h.HA}VK00037
99866	119076	y[1] w[1118]; PBac{y[+mDint2] w[+mC]=UAS-yellow-h.HA}VK00033
99867	119077	y[1] w[1118]; PBac{y[+mDint2] w[+mC]=UAS-Gat.HA}VK00037
99868	119078	y[1] w[1118]; PBac{y[+mDint2] w[+mC]=UAS-Gat.HA}VK00033
99869	119079	y[1] w[1118]; PBac{y[+mDint2] w[+mC]=UAS-CG1909.HA}VK00037
99870	119080	y[1] w[1118]; PBac{y[+mDint2] w[+mC]=UAS-CG1909.HA}VK00033
99871	119081	y[1] w[1118]; PBac{y[+mDint2] w[+mC]=UAS-CGPolr1G.HA}VK00037
99872	119082	y[1] w[1118]; PBac{y[+mDint2] w[+mC]=UAS-CGPolr1G.HA}VK00033
99873	119083	y[1] w[1118]; PBac{y[+mDint2] w[+mC]=UAS-Mpv17.HA}VK00037
99874	119084	y[1] w[1118]; PBac{y[+mDint2] w[+mC]=UAS-Mpv17.HA}VK00033
99875	119085	y[1] w[1118]; PBac{y[+mDint2] w[+mC]=UAS-dpr7.HA}VK00037
99876	119086	y[1] w[1118]; PBac{y[+mDint2] w[+mC]=UAS-dpr7.HA}VK00033
99877	119087	y[1] w[1118]; PBac{y[+mDint2] w[+mC]=UAS-CG33521.HA}VK00037
99878	119088	y[1] w[1118]; PBac{y[+mDint2] w[+mC]=UAS-CG33521.HA}VK00033
99879	119089	y[1] w[1118]; PBac{y[+mDint2] w[+mC]=UAS-CG33941.HA}VK00037
99880	119090	y[1] w[1118]; PBac{y[+mDint2] w[+mC]=UAS-CG33941.HA}VK00033
603548	119194	y[1] w[1118]; PBac{y[+mDint2] w[+mC]=UAS-zfh2.HA}VK00037
603549	119195	y[1] w[1118]; PBac{y[+mDint2] w[+mC]=UAS-zfh2.HA}VK00033
603550	119196	y[1] w[1118]; PBac{y[+mDint2] w[+mC]=UAS-Slip1.HA}VK00037
603551	119197	y[1] w[1118]; PBac{y[+mDint2] w[+mC]=UAS-Slip1.HA}VK00033
603552	119198	y[1] w[1118]; PBac{y[+mDint2] w[+mC]=UAS-CG31998.HA}VK00037
603553	119199	y[1] w[1118]; PBac{y[+mDint2] w[+mC]=UAS-CG31998.HA}VK00033
603554	119200	y[1] w[1118]; PBac{y[+mDint2] w[+mC]=UAS-PMCA.HA}VK00037
603555	119201	y[1] w[1118]; PBac{y[+mDint2] w[+mC]=UAS-PMCA.HA}VK00033
606981		y[1] w[*]; P{y[+t*]=UAS-Glu-RA.R}2
606982		y[1] w[*]; P{y[+t*]=UAS-Glu-RA.R}3
**3B: UAS.human cDNA**		**Genotype**
	118978	w[1118]; PBac{y[+mDint2] w[+mC]=UAS-hLIMD2.HA}VK00033
	118979	w[1118]; PBac{y[+mDint2] w[+mC]=UAS-hRNF6.HA}VK00033
	118980	w[1118]; PBac{y[+mDint2] w[+mC]=UAS-hTNRC6C.HA}VK00033
	118982	w[1118]; PBac{y[+mDint2] w[+mC]=UAS-hSLC39A8.HA.N}VK00033
	118983	w[1118]; PBac{y[+mDint2] w[+mC]=UAS-hTAF3.HA}VK00033
	118984	w[1118]; PBac{y[+mDint2] w[+mC]=UAS-hMEX3C.HA}VK00033
	118985	w[1118]; PBac{y[+mDint2] w[+mC]=UAS-hFBLN2.HA}VK00033
	118986	w[1118]; PBac{y[+mDint2] w[+mC]=UAS-hNDUFS1.HA}VK00033
	118987	w[1118]; PBac{y[+mDint2] w[+mC]=UAS-hTGFB3.N}VK00033
	118988	w[1118]; PBac{y[+mDint2] w[+mC]=UAS-hTGFB2.N}VK00033
	118989	w[1118]; PBac{y[+mDint2] w[+mC]=UAS-hTGFB1.N}VK00033
	118990	w[1118]; PBac{y[+mDint2] w[+mC]=UAS-hINHBA.N}VK00033
	118991	w[1118]; PBac{y[+mDint2] w[+mC]=UAS-hINHBB.N}VK00033
	118992	w[1118]; PBac{y[+mDint2] w[+mC]=UAS-hINHBC.N}VK00033
	118993	w[1118]; PBac{y[+mDint2] w[+mC]=UAS-hINHBE.N}VK00033
	118994	w[1118]; PBac{y[+mDint2] w[+mC]=UAS-hZFHX2.HA}VK00033
	118995	w[1118]; PBac{y[+mDint2] w[+mC]=UAS-hZFHX3.HA}VK00033

	118996	w[1118]; PBac{y[+mDint2] w[+mC]=UAS-hONECUT1.HA}VK00033
	118997	w[1118]; PBac{y[+mDint2] w[+mC]=UAS-hTNRC6A.HA}VK00037
98424	118998	w[1118]; PBac{y[+mDint2] w[+mC]=UAS-hLIMD2.HA}VK00037
98425	118999	w[1118]; PBac{y[+mDint2] w[+mC]=UAS-hRNF6.HA}VK00037
98426	119000	w[1118]; PBac{y[+mDint2] w[+mC]=UAS-hTNRC6C.HA}VK00037
	119001	w[1118]; PBac{y[+mDint2] w[+mC]=UAS-hFBN2.N}VK00033
98429	119003	w[1118]; PBac{y[+mDint2] w[+mC]=UAS-hTAF3.N}VK00037
98430	119004	w[1118]; PBac{y[+mDint2] w[+mC]=UAS-hMEX3C.HA}VK00037
98431	119005	w[1118]; PBac{y[+mDint2] w[+mC]=UAS-hFBLN2.HA}VK00037
98434	119006	w[1118]; PBac{y[+mDint2] w[+mC]=UAS-hONECUT1.HA}VK00037
600291	119016	w[1118]; PBac{y[+mDint2] w[+mC]=UAS-hLIMA1.N}VK00037
600292	119017	w[1118]; PBac{y[+mDint2] w[+mC]=UAS-hTCF7L1.HA}VK00037
600293	119022	w[1118]; PBac{y[+mDint2] w[+mC]=UAS-hGLI2.N}VK00037
600294	119019	w[1118]; PBac{y[+mDint2] w[+mC]=UAS-hSYT5.HA}VK00037
600295	119020	w[1118]; PBac{y[+mDint2] w[+mC]=UAS-hINHA.N}VK00037
600296	119024	w[1118]; PBac{y[+mDint2] w[+mC]=UAS-hZYX.HA}VK00037
602373	119018	w[1118]; PBac{y[+mDint2] w[+mC]=UAS-hCADPS2.N}VK00037
602374	119021	w[1118]; PBac{y[+mDint2] w[+mC]=UAS-hARHGAP36.HA}VK00037
602375	119023	w[1118]; PBac{y[+mDint2] w[+mC]=UAS-hTRIP6.HA}VK00037
602376	119002	w[1118]; PBac{y[+mDint2] w[+mC]=UAS-hSLC39A8.HA.N}VK00037
602377	119006	w[1118]; PBac{y[+mDint2] w[+mC]=UAS-hONECUT1.HA}VK00037
602451	119138	w[1118]; PBac{y[+mDint2] w[+mC]=UAS-hMSTN.N}VK00033
602452	119139	w[1118]; PBac{y[+mDint2] w[+mC]=UAS-hGRM1.HA}VK00033
602453	119140	w[1118]; PBac{y[+mDint2] w[+mC]=UAS-hDYRK1A.HA}VK00033
602454	119141	w[1118]; PBac{y[+mDint2] w[+mC]=UAS-hTDG.HA}VK00033
602455	119142	w[1118]; PBac{y[+mDint2] w[+mC]=UAS-hPLXNB2.N}VK00033
602456	119143	w[1118]; PBac{y[+mDint2] w[+mC]=UAS-hPLXNB3.N}VK00033
	119144	w[1118]; PBac{y[+mDint2] w[+mC]=UAS-hMSTN.N}VK00037
	119145	w[1118]; PBac{y[+mDint2] w[+mC]=UAS-hGRM1.HA}VK00037
	119146	w[1118]; PBac{y[+mDint2] w[+mC]=UAS-hDYRK1A.HA}VK00037
	119147	w[1118]; PBac{y[+mDint2] w[+mC]=UAS-hTDC.HA}VK00037
	119148	w[1118]; PBac{y[+mDint2] w[+mC]=UAS-hPLXNB2.N}VK00037
	119149	w[1118]; PBac{y[+mDint2] w[+mC]=UAS-hPLXNB3.N}VK00037
606870		w[1118]; PBac{y[+mDint2] w[+mC]=UAS-hGDF11.N}VK00033
606871	118981	y[1] w[*]; PBac{y[+mDint2] w[+mC]=UAS-hFBN2.N}VK00033
607313	119774	w[1118]; PBac{y[+mDint2] w[+mC]=UAS-hATP12A.HA}VK00037
607314	119776	w[1118]; PBac{y[+mDint2] w[+mC]=UAS-hEIF4G1.HA}VK00037/CyO
607315	119778	w[1118]; PBac{y[+mDint2] w[+mC]=UAS-hFOXB1.HA}VK00037
607316	119780	w[1118]; PBac{y[+mDint2] w[+mC]=UAS-hGIGYF1.HA}VK00037
607317	119782	w[1118]; PBac{y[+mDint2] w[+mC]=UAS-hGLI3.HA}VK00037
607318	119784	w[1118]; PBac{y[+mDint2] w[+mC]=UAS-hGRIK1.HA}VK00037/CyO
607319	119786	w[1118]; PBac{y[+mDint2] w[+mC]=UAS-hMED26.HA}VK00037
607320	119788	w[1118]; PBac{y[+mDint2] w[+mC]=UAS-hPURA.HA}VK00037
607321	119790	w[1118]; PBac{y[+mDint2] w[+mC]=UAS-hYY2.HA}VK00037
607322	119792	w[1118]; PBac{y[+mDint2] w[+mC]=UAS-hZNF362.HA}VK00037
607323	119794	w[1118]; PBac{y[+mDint2] w[+mC]=UAS-hZNF384.HA}VK00037
	119775	w[1118]; PBac{y[+mDint2] w[+mC]=UAS-hATP12A.HA}VK00033
	119777	w[1118]; PBac{y[+mDint2] w[+mC]=UAS-hEIF4G1.HA}VK00033
	119779	w[1118]; PBac{y[+mDint2] w[+mC]=UAS-hFOXB1.HA}VK00033

	119781	w[1118]; PBac{y[+mDint2] w[+mC]=UAS-hGIGYF1.HA}VK00033
	119783	w[1118]; PBac{y[+mDint2] w[+mC]=UAS-hGLI3.HA}VK00033
	119785	w[1118]; PBac{y[+mDint2] w[+mC]=UAS-hGRIK1.HA}VK00033
	119787	w[1118]; PBac{y[+mDint2] w[+mC]=UAS-hMED26.HA}VK00033
	119789	w[1118]; PBac{y[+mDint2] w[+mC]=UAS-hPURA.HA}VK00033
	119791	w[1118]; PBac{y[+mDint2] w[+mC]=UAS-hYY2.HA}VK00033/ TM3, Sb[1]
	119793	w[1118]; PBac{y[+mDint2] w[+mC]=UAS-hZNF362.HA}VK00033
	119795	w[1118]; PBac{y[+mDint2] w[+mC]=UAS-hZNF384.HA}VK00033

Table S4

FCRP: Update to expression verification for Bloomington HA-tagged stocks			
**4A: UAS.fly cDNA**			
**UFO clones in red being replaced by pGW-HA.attB**	**BL9752 attP-VK37 chromosome II**	**BL9750 attP-VK33 chromosome III**	**Antibody concentration Adult eye phenotype?**
**4E-T** UFO clone	Not Detected	HA tag expression	1:100 - no
**Arf4** UFO clone	Not Detected	Not Detected	1:100 - no
**Arl4**	HA tag expression	HA tag expression	1:500 - no
**Cals** UFO clone	Not Detected	Not Detected	1:500 - no
**CaMKI**	HA tag expression	HA tag expression	1:500 - no
**CG11076/Polr1G**	HA tag expression	HA tag expression	1:500 - no
**CG11077/Mpv17**	HA tag expression	HA tag expression	1:500 - no
**CG11155** UFO clone	Not Detected	Not Detected	1:100 - no
**CG1674**	HA tag expression	HA tag expression	1:500 - no
**CG1909**	HA tag expression	HA tag expression	1:500 - no
**CG2316/Abcd1** UFO clone	HA tag expression	Not Detected	1:100 - no
**CG31997** UFO clone	Not Detected	Not Detected	1:100 - no
**CG31998**	HA tag expression	HA tag expression	1:500 - no
**CG31999** UFO clone	Not Detected	Not Detected	1:100 - no
**CG32017** UFO clone	Not Detected	Not Detected	1:100 - no
**CG32850/Rnf11** UFO clone	Not Detected	Not Detected	1:100 - no
**CG33521**	HA tag expression	HA tag expression	1:500 - no
**CG33941**	HA tag expression	HA tag expression	1:500 - no
**Dpr7**	HA tag expression	HA tag weak	1:500 - no
**Dyrk3**	HA tag expression	HA tag expression	1:500 - no
**Ekar** UFO clone	Not Detected	Not Detected	1:100 - no
**Gat**	HA tag expression	Not Detected	1:500 - no
**Kif3C**	Not Detected[a]	Not Detected[a]	1:100 - no
**Lgs**	HA tag expression	HA tag expression	1:500 - no
**ND-49**	HA tag expression	HA tag expression	1:500 - no
**Onecut**	N/A	HA tag expression[b]	1:500 - glazed eye
**Pho**	N/A	HA tag expression[b]	1:500 - rough eye
**PMCA**	HA tag expression	HA tag expression	1:500 - no
**Slip1**	HA tag expression	HA tag expression	1:500 - no
**Yellow-h**	HA tag expression	HA tag expression	1:500 - no
**Zfh2**	HA tag expression	HA tag expression	1:500 - rough eye

a. *kif3C* transcript regulated by *miR-184* leading to no protein expression (Iovino et al. 2009)
b. UAS.ORFeome stocks in the same transgene on chromosome III in attP-ZH-86Fb (Bischof et al. 2013).

Table S4 (continued)

FCRP: Update to expression verification for Bloomington HA-tagged stocks			
**4B: UAS.human cDNA**			
**One to be replaced in red**	**BL9752 attP-VK37 chromosome II**	**BL9750 attP-VK33 chromosome III**	**Antibody concentration Adult eye phenotype?**
**ARHGAP36**	HA tag expression	not tested	1:500 - no
**ARL4A**	HA tag expression	HA tag expression	1:500 - no
**ATP12A**	not tested	HA tag expression	1:500 - no
**DYRK1A**	not tested	Not Detected	1:100 - no
**EIF4G1**	HA tag expression	HA tag expression	1:500 - no
**FBLN2**	HA tag expression	not tested	1:500 - no
**FOXB1**	HA tag expression	HA tag expression	1:500 - rough eye
**FOXB2**	HA tag expression	HA tag expression	1:500 - rough eye
**GIGYFI**	HA tag expression	HA tag expression	1:500 - no
**GLI3**	HA tag expression	HA tag expression	1:500 - no
**GRIKI**	HA tag expression	HA tag expression	1:500 - no
**GRM1**	not tested	HA tag expression	1:500 - rough eye
**LIMD2**	HA tag expression	not tested	1:500 - no
**MED26**	HA tag expression	HA tag expression	1:500 - no
**MEX3C**	HA tag expression	not tested	1:500 - no
**NDUFS1**	HA tag expression	not tested	1:500 - no
**ONECUT1**	HA tag expression	not tested	1:500 - pharate lethal; escaper small glazed eye
**PURA**	HA tag expression	pipeline	1:500 - pharate lethal; escaper rough eye
**RNF6**	HA tag weak	not tested	1:100 - no
**SKOR1**	HA tag expression	HA tag expression	1:500 - no
**SKOR2**	HA tag expression	HA tag expression	1:500 - rough eye
**SLC39A8**	HA tag expression	not tested	1:500 - no
**SYT5**	HA tag expression	not tested	1:500 - no
**TDG**	not tested	HA tag expression	1:500 - no
**TNRC6C**	HA tag expression	not tested	1:500 - no
**TRIP6**	HA tag expression	not tested	1:500 - no
**VPS72**	HA tag expression	not tested	1:500 - no
**YY1**	HA tag expression	HA tag expression	1:500 - rough eye
**YY2**	HA tag expression	HA tag expression	1:500 - rough eye
**ZFHX2**	N/A	HA tag expression[a]	1:100 - no
**ZFHX3**	N/A	HA tag expression[a]	1:500 - no
**ZNF362**	HA tag expression	HA tag expression	1:500 - pharate lethal; escaper glazed eye
**ZNF384**	HA tag expression	HA tag expression	1:500 - glazed eye
**ZYX**	HA tag expression	not tested	1:500 - no

a. Same transgene inserted on chromosome III in attP2 (BL8622).

Table S5

5A. FCRP: New FRT101F Tub.GAL80-102B & -102C stocks with Bloomington & Kyoto stock numbers		
**Bloomington**	**Kyoto**	**Genotype**
		**With and without the Ds-Red transformation marker**
606068	119730	w[1118]; TI{TI}FRT101F TI{RFP[DsRed.3xP3.cUa]=Tub-GAL80.W}102BDsRed+/ TI{GMR-HMS04515}Gat[eya]
606803		w[1118]; TI{TI}FRT101F TI{Tub-GAL80.W}102B/ In(4)ci[D], ci[D] pan[ciD]
606069	119731	w[1118]; TI{TI}FRT101F TI{RFP[DsRed.3xP3.cUa]=Tub-GAL80.W}102CDsRed+/ TI{GMR-HMS04515}Gat[eya]
606983		w[1118]; TI{TI}FRT101F TI{Tub-GAL80.W}102C/ In(4)ci[D], ci[D] pan[ciD]
**5B. All FRT101F CRISPR mutants in protein coding genes (see Weasner et al. 2025 for details)**		
600261	119072	y[1] w[1118]; P{w[+mC]=Act5C.GAL4}25FO1, P{w[+mC]=UAS-GFP.U}2/SM6a; TI{TI}FRT101F zfh2[51A]/TI{GMR-HMS04515}Gat[eya]
600262	119073	y[1] w[1118]; TI{TI}FRT101F apolpp[B]/In(4)ci[D], ci[D] pan[ciD]
600263	119074	y[1] w[1118]; TI{TI}FRT101F Pur-alpha[B]/In(4)ci[D], ci[D] pan[ciD]
602194	119160	y[1] w[1118]; TI{TI}FRT101F Ank[A]
602195	119161	y[1] w[1118]; TI{TI}FRT101F ey[B]/In(4)ci[D], ci[D] pan[ciD]
602196	119162	y[1] w[1118]; TI{TI}FRT101F Ephrin[A]
602197	119163	y[1] w[1118]; TI{TI}FRT101F anne[B]/In(4)ci[D], ci[D] pan[ciD]
602198	119164	y[1] w[1118]; TI{TI}FRT101F yellow-h[A]
602199	119165	y[1] w[1118]; TI{TI}FRT101F Abcd1[A]
602200	119166	y[1] w[1118]; TI{TI}FRT101F RhoGAP102A[A]
602201	119167	y[1] w[1118]; TI{TI}FRT101F Rad23[8]
602202	119168	y[1] w[1118]; TI{TI}FRT101F mav[B]
602203	119169	y[1] w[1118]; TI{TI}FRT101F 4E-T[A]
602204	119170	y[1] w[1118]; TI{TI}FRT101F Hcf[C]/In(4)ci[D], ci[D] pan[ciD]
602205	119171	y[1] w[1118]; TI{TI}FRT101F pan[F]/P{w[+mC]=ActGFP}unc-13[GJ]
603543	119189	y[1] w[1118]; TI{TI}FRT101F Taf3[A]/In(4)ci[D], ci[D] pan[ciD]
603544	119190	y[1] w[1118]; TI{TI}FRT101F toy[C]/In(4)ci[D], ci[D] pan[ciD]
603545	119191	y[1] w[1118]; TI{TI}FRT101F Syt7[B]/In(4)ci[D], ci[D] pan[ciD]
603546	119192	y[1] w[1118]; TI{TI}FRT101F Sox102F[C]
603547	119193	y[1] w[*]; TI{TI}FRT101F bt[A]/TI{GMR-HMS04515}Gat[eya]
605312	119261	y[1] w[1118]; TI{TI}FRT101F anne[D]/In(4)ci[D], ci[D] pan[ciD]
605313	119262	y[1] w[1118]; TI{TI}FRT101F apolpp[J]/In(4)ci[D], ci[D] pan[ciD]
605314	119263	y[1] w[1118]; TI{TI}FRT101F ATPsynbeta[C]/In(4)ci[D], ci[D] pan[ciD]
605315	119264	y[1] w[1118]; TI{TI}FRT101F CaMKI[F]
605316	119265	y[1] w[1118]; TI{TI}FRT101F CG11360[D]
605317	119266	y[1] w[1118]; TI{TI}FRT101F CG33521[C]
605318	119267	y[1] w[1118]; TI{TI}FRT101F ci[C]/P{w[+mC]=ActGFP}unc-13[GJ]
605319	119268	y[1] w[1118]; TI{TI}FRT101F Eph[G]
605320	119269	y[1] w[1118]; TI{TI}FRT101F ey[A]/In(4)ci[D], ci[D] pan[ciD]
605321	119270	y[1] w[1118]; TI{TI}FRT101F Gyf[B]/In(4)ci[D], ci[D] pan[ciD]
605322	119271	y[1] w[*]; TI{TI}FRT101F Jyalpha[I]/In(4)ci[D], ci[D] pan[ciD]
605323	119272	y[1] w[*]; TI{TI}FRT101F Jyalpha[P]/TI{GMR-HMS04515}Gat[eya]
605324	119273	y[1] w[1118]; TI{TI}FRT101F Kif3C[A]/In(4)ci[D], ci[D] pan[ciD]
605325	119274	y[1] w[1118]; TI{TI}FRT101F onecut[G]
605326	119275	y[1] w[1118]; TI{TI}FRT101F pan[I]/P{w[+mC]=ActGFP}unc-13[GJ]
605327	119276	y[1] w[1118]; TI{TI}FRT101F PMCA[D]/In(4)ci[D], ci[D] pan[ciD]
605328	119277	y[1] w[1118]; TI{TI}FRT101F Rad23[3]

605329	119278	y[1] w[1118]; TI{TI}FRT101F Rnf11[A]
605330	119279	y[1] w[1118]; TI{TI}FRT101F Rnf11[F]
605331	119280	y[1] w[1118]; TI{TI}FRT101F Tdg[C]/In(4)ci[D], ci[D] pan[ciD]
605332	119281	y[1] w[1118]; TI{TI}FRT101F toy[A]/In(4)ci[D], ci[D] pan[ciD]
605333	119282	y[1] w[1118]; TI{TI}FRT101F yellow-h[D]
605334	119283	y[1] w[1118]; TI{TI}FRT101F zfh2[D]/In(4)ci[D], ci[D] pan[ciD]
605780	119687	y[1] w[1118]; TI{TI}FRT101F 4E-T[D]
605781	119688	y[1] w[1118]; TI{TI}FRT101F Actbeta[B]/In(4)ci[D], ci[D] pan[ciD]
605782	119689	y[1] w[1118]; TI{TI}FRT101F Arf4[A]/In(4)ci[D], ci[D] pan[ciD]
605783	119690	y[1] w[1118]; TI{TI}FRT101F CamKI[I]
605784	119691	y[1] w[1118]; TI{TI}FRT101F CG1674[E]
605785	119692	y[1] w[1118]; TI{TI}FRT101F CG1909[B]
605786	119693	y[1] w[1118]; TI{TI}FRT101F CG31998[C]/In(4)ci[D], ci[D] pan[ciD]
605787	119694	y[1] w[1118]; TI{TI}FRT101F CG31998[D]/In(4)ci[D], ci[D] pan[ciD]
605788	119695	y[1] w[1118]; TI{TI}FRT101F CG32017[E]/In(4)ci[D], ci[D] pan[ciD]
605789	119696	y[1] w[1118]; TI{TI}FRT101F CG32017[K]/In(4)ci[D], ci[D] pan[ciD]
605790	119697	y[1] w[1118]; TI{TI}FRT101F Crk[E]/In(4)ci[D], ci[D] pan[ciD]
605791	119698	y[1] w[1118]; TI{TI}FRT101F Eph[A]
605792	119699	y[1] w[1118]; TI{TI}FRT101F fd102c[D]
605793	119700	y[1] w[1118]; TI{TI}FRT101F Gyf[F]/In(4)ci[D], ci[D] pan[ciD]
605794	119701	y[1] w[1118]; TI{TI}FRT101F mav[D]
605795	119702	y[1] w[1118]; TI{TI}FRT101F mGluR[B]
605796	119703	y[1] w[1118]; TI{TI}FRT101F mGluR[E]
605797	119704	y[1] w[1118]; TI{TI}FRT101F onecut[H]
605798	119705	y[1] w[1118]; TI{TI}FRT101F pho[F]/In(4)ci[D], ci[D] pan[ciD]
605799	119706	y[1] w[1118]; TI{TI}FRT101F RhoGAP102A[E]
605800	119707	y[1] w[1118]; TI{TI}FRT101F sv[F]/In(4)ci[D], ci[D] pan[ciD]
605801	119708	y[1] w[1118]; TI{TI}FRT101F Tdg[F]
605802	119709	y[1] w[1118]; TI{TI}FRT101F zfh2[A]/In(4)ci[D], ci[D] pan[ciD]
	119284	y[1] w[1118]; TI{TI}FRT101F JYalpha[L]
	119285	y[1] w[1118]; TI{TI}FRT101F ci[F]
	119286	y[1] w[1118]; TI{TI}FRT101F Arl4[A]
	119287	y[1] w[1118]; TI{TI}FRT101F Crk[G]
	119288	y[1] w[1118]; TI{TI}FRT101F CG31999[C]
	119289	y[1] w[1118]; TI{TI}FRT101F CG1674[C]
	119290	y[1] w[1118]; TI{TI}FRT101F dpr7[A]
	119291	y[1] w[1118]; TI{TI}FRT101F RhoGAP102A[B]
	119292	y[1] w[1118]; TI{TI}FRT101F Syt7[D]
	119293	y[1] w[1118]; TI{TI}FRT101F Rad23[B]
	119294	y[1] w[1118]; TI{TI}FRT101F Hcf[A]/In(4)ci[D], ci[D] pan[ciD]
	119295	y[1] w[1118]; TI{TI}FRT101F lgs[C]
	119296	y[1] w[1118]; TI{TI}FRT101F Taf3[F]
	119297	y[1] w[1118]; TI{TI}FRT101F Tdg[I]
	119298	y[1] w[1118]; TI{TI}FRT101F ND-49 [A]
	119299	y[1] w[1118]; TI{TI}FRT101F CG1909[A]
	119300	y[1] w[1118]; TI{TI}FRT101F Ekar[C]
	119301	y[1] w[1118]; TI{TI}FRT101F Slip1 [A]/In(4)ci[D], ci[D] pan[ciD]
	119302	y[1] w[1118]; TI{TI}FRT101F myo[A]
	119303	y[1] w[1118]; TI{TI}FRT101F bt[C]
	119304	y[1] w[1118]; TI{TI}FRT101F MED26[A]

	119305	y[1] w[1118]; TI{TI}FRT101F Sox102F[B]
	119306	y[1] w[1118]; TI{TI}FRT101F unc-13[C]/TI{GMR-HMS04515}Gat[eya]
	119307	y[1] w[1118]; TI{TI}FRT101F toy[B]/In(4)ci[D], ci[D] pan[ciD]
	119308	y[1] w[1118]; TI{TI}FRT101F Actbeta[A]
	119309	y[1] w[1118]; TI{TI}FRT101F sv[F]/In(4)ci[D], ci[D] pan[ciD]
	119310	y[1] w[1118]; TI{TI}FRT101F ukar[D]
	119311	y[1] w[1118]; TI{TI}FRT101F CG32017[B]
605962	119710	y[1] w[1118]; TI{TI}FRT101F PlexB[B]/In(4)ci[D], ci[D] pan[ciD]
605963	119711	y[1] w[1118]; TI{TI}FRT101F CG33978[B]/In(4)ci[D], ci[D] pan[ciD]
605964	119712	y[1] w[1118]; TI{TI}FRT101F Abcd1[K]/In(4)ci[D], ci[D] pan[ciD]
605965	119713	y[1] w[1118]; TI{TI}FRT101F CG31999[A]/In(4)ci[D], ci[D] pan[ciD]
605966	119714	y[1] w[1118]; TI{TI}FRT101F Nfl[B]/In(4)ci[D], ci[D] pan[ciD]
605967	119715	y[1] w[1118]; TI{TI}FRT101F Nfl[E]/In(4)ci[D], ci[D] pan[ciD]
605968	119716	y[1] w[1118]; TI{TI}FRT101F lgs[E]/In(4)ci[D], ci[D] pan[ciD]
605969	119717	y[1] w[1118]; TI{TI}FRT101F myo[C]/In(4)ci[D], ci[D] pan[ciD]
605970	119718	y[1] w[1118]; TI{TI}FRT101F eIF4G1[B]/In(4)ci[D], ci[D] pan[ciD]
605971	119719	y[1] w[1118]; TI{TI}FRT101F eIF4G1[E]/In(4)ci[D], ci[D] pan[ciD]
605972	119720	y[1] w[1118]; TI{TI}FRT101F Zyx[D]/In(4)ci[D], ci[D] pan[ciD]
605973	119721	y[1] w[1118]; TI{TI}FRT101F sv[B]/In(4)ci[D], ci[D] pan[ciD]
605974	119722	y[1] w[1118]; TI{TI}FRT101F Cadps[B]/In(4)ci[D], ci[D] pan[ciD]
605975	119723	y[1] w[1118]; TI{TI}FRT101F CG1674[A]
605976	119724	y[1] w[*]; TI{TI}FRT101F Ank[J]
605977	119725	y[1] w[*]; TI{TI}FRT101F PMCA[B]/TI{GMR-HMS04515}Gat[eya]
605978	119726	y[1] w[*]; TI{TI}FRT101F fuss[A]/TI{GMR-HMS04515}Gat[eya]
605979	119727	y[1] w[*]; TI{TI}FRT101F fuss[C]
605980	119728	y[1] w[*]; TI{TI}FRT101F Mitf[E]/TI{GMR-HMS04515}Gat[eya]
605981	119729	y[1] w[*]; TI{TI}FRT101F unc-13[E]/TI{GMR-HMS04515}Gat[eya
606072	119738	y[1] w[1118]; TI{TI}FRT101F CG33978[A]/In(4)ci[D], ci[D] pan[ciD]
606073	119739	y[1] w[1118]; TI{TI}FRT101F Arl4[D]
606074	119740	y[1] w[1118]; TI{TI}FRT101F CG31999[G]/In(4)ci[D], ci[D] pan[ciD]
606075	119741	y[1] w[1118]; TI{TI}FRT101F dpr7[B]
606076	119742	y[1] w[1118]; TI{TI}FRT101F dpr7[G]
606077	119743	y[1] w[1118]; TI{TI}FRT101F dati[B]/In(4)ci[D], ci[D] pan[ciD]
606078	119744	y[1] w[1118]; TI{TI}FRT101F Asator[A]/In(4)ci[D], ci[D] pan[ciD]
606079	119745	y[1] w[1118]; TI{TI}FRT101F Asator[F]/In(4)ci[D], ci[D] pan[ciD]
606080	119746	y[1] w[1118]; TI{TI}FRT101F ND-49[B]/In(4)ci[D], ci[D] pan[ciD]
606081	119747	y[1] w[1118]; TI{TI}FRT101F ND-49[G]/In(4)ci[D], ci[D] pan[ciD]
606082	119748	y[1] w[*]; TI{TI}FRT101F Ephrin[H]/TI{GMR-HMS04515}Gat[eya]
606083	119749	y[1] w[1118]; TI{TI}FRT101F Ekar[B]
606084	119750	y[1] w[1118]; TI{TI}FRT101F Ekar[I]
606085	119751	y[1] w[1118]; TI{TI}FRT101F PlexA[D]/In(4)ci[D], ci[D] pan[ciD]
606086	119752	y[1] w[1118]; TI{TI}FRT101F apolpp[N]/In(4)ci[D], ci[D] pan[ciD]
606087	119753	y[1] w[1118]; TI{TI}FRT101F ukar[B]
606088	119754	y[1] w[1118]; TI{TI}FRT101F ukar[C]
606089	119755	y[1] w[1118]; TI{TI}FRT101F Kif3C[C]
606090	119756	y[1] w[1118]; TI{TI}FRT101F Kif3C[E]
606091	119757	y[1] w[1118]; TI{TI}FRT101F PIP4K[B]/In(4)ci[D], ci[D] pan[ciD]
606092	119758	y[1] w[1118]; TI{TI}FRT101F PIP4K[C]/In(4)ci[D], ci[D] pan[ciD]
606093	119759	y[1] w[*]; TI{TI}FRT101F CG33521[A]/TI{GMR-HMS04515}Gat[eya]
606094	119760	y[1] w[1118]; TI{TI}FRT101F Dyrk3[B]

606095	119761	y[1] w[1118]; TI{TI}FRT101F Dyrk3[N]
	119796	y[1] w[1118]; TI{TI}FRT101F Abcd1[G]
	119797	y[1] w[1118]; TI{TI}FRT101F fd102c[E]
	119798	y[1] w[1118]; TI{TI}FRT101F Polr1G[A]
	119799	y[1] w[1118]; TI{TI}FRT101F Cals[F]
	119800	y[1] w[1118]; TI{TI}FRT101F eIF4G1[D]
	119801	y[1] w[1118]; TI{TI}FRT101F CG31997[I]
	119802	y[1] w[*]; TI{TI}FRT101F Nfl[G]
	119803	y[1] w[*]; TI{TI}FRT101F CamKI[G] / TI{GMR-HMS04515}Gat[eya]
	119804	y[1] w[*]; TI{TI}FRT101F Ephrin [F] / TI{GMR-HMS04515}Gat[eya]
	119805	y[1] w[*]; TI{TI}FRT101F onecut[A] / TI{GMR-HMS04515}Gat[eya]
	119806	y[1] w[*]; TI{TI}FRT101F mav[A] / TI{GMR-HMS04515}Gat[eya]
	119807	y[1] w[1118]; TI{TI}FRT101F Plex A[B] / In(4)ci[D], ci[D] pan[ciD]
	119808	y[1] w[*]; TI{TI}FRT101F Mpv17[D] / TI{GMR-HMS04515}Gat[eya]
	119809	y[1] w[*]; TI{TI}FRT101F CG33521[D] / TI{GMR-HMS04515}Gat[eya]
	119810	y[1] w[*]; TI{TI}FRT101F PIP4K[G] / TI{GMR-HMS04515}Gat[eya]
	119811	y[1] w[1118]; TI{TI}FRT101F Dyrk3[R]
	119812	y[1] w[*]; TI{TI}FRT101F Cadps[J] / TI{GMR-HMS04515}Gat[eya]
	119813	y[1] w[1118]; TI{TI}FRT101F Mitf[P]
	119814	y[1] w[1118]; TI{TI}FRT101F Kif3C [I]
	119815	y[1] w[*]; TI{TI}FRT101F CG11360[A]
	119816	y[1] w[*]; TI{TI}FRT101F anne[R]
	119817	y[1] w[*]; TI{TI}FRT101F fuss[N] / TI{GMR-HMS04515}Gat[eya]
	119818	y[1] w[*]; TI{TI}FRT101F Gyf[I] / TI{GMR-HMS04515}Gat[eya]
	119819	y[1] w[*]; TI{TI}FRT101F CamKII[K] / TI{GMR-HMS04515}Gat[eya]
	119820	y[1] w[*]; TI{TI}FRT101F Rps3A[A]
	119821	y[1] w[*]; TI{TI}FRT101F Gat[C]
	119822	y[1] w[*]; TI{TI}FRT101F CG32006[A]
606722	119732	y[1] w[*]; TI{TI}FRT101F Slip1[D]
606723	119733	y[1] w[*]; TI{TI}FRT101F Slip1[H]
606724	119734	y[1] w[*]; TI{TI}FRT101F CG11360[I]
606725	119735	y[1] w[*]; TI{TI}FRT101F myo[D]/TI{GMR-HMS04515}Gat[eya]
606726	119736	y[1] w[1118]; TI{TI}FRT101F CamKII[H]/In(4)ci[D], ci[D] pan[ciD]
606727	119737	y[1] w[1118]; TI{TI}FRT101F Mitf[F]/In(4)ci[D], ci[D] pan[ciD]
606864	119762	y[l] w(*]; TI{TI}FRT101F CG31997[P]
606865	119763	y[l] w(*]; TI{TI}FRT101F MED26[P]/TI{GMR-HMS04515}Gat[eya]
606866	119764	y[l] w(*]; TI{TI}FRT101F MED26[L]/TI{GMR-HMS04515}Gat[eya]
606867	119765	y[l] w(*]; Df(4)Mpv17[L], TI{TI}FRT101F Mpv17[L] asRNA:CR44031(L]
606868	119766	y[l] w(*]; Df(4)Mpv17[T], TI{TI}FRT101F Mpv17[T] asRNA:CR44031(T]
606869	119767	y[l] w(*]; TI{TI}FRT101F CaMKII[L]
606933	119768	y[1] w[*]; TI{TI}FRT101F anne[K]/TI{GMR-HMS04515}Gat[eya]
607324	119823	y[1] w[*]; TI{TI}FRT101F CG33941[A]
607325	119824	y[1] w[*]; TI{TI}FRT101F CG33941[B]
607326	119825	y[1] w[*]; TI{TI}FRT101F Gat[A]/In(4)ci[D], ci[D] pan[ciD]
607327	119826	y[1] w[*]; TI{TI}FRT101F Gat[B]/In(4)ci[D], ci[D] pan[ciD]
607328	119827	y[1] w[*]; TI{TI}FRT101F CG32006[B]/In(4)ci[D], ci[D] pan[ciD]
607329	119828	y[1] w[*]; TI{TI}FRT101F Cals[L]
607330	119829	y[1] w[*]; TI{TI}FRT101F Cals[R]

Table S6

FCRP & GDP: Update to coverage for all fourth coding genes in 5 sets (FCRP in red & GDP in black)						
**Genes proximal to distal long arm**	**Flybase gene ID**	**UAS.fly cDNA chromosome**	**T2A.GAL4 gene traps lethal/viable**	**eGFP protein traps lethal/viable**	**UAS.human cDNA ortholog names**	**New mutants FRT101F number; truncation lethal-viable; tested clone allele**
Jyalpha	FBgn0267363	pipeline	lethal	lethal	ATP12A	3 mutants; lethal
PlexB	FBgn0052009	II/III	lethal	viable	PLXB2, PLXB3	1 mutant; lethal
ci	FBgn0265633	II/III	lethal	viable	GLI2. GLI3	2 mutants; lethal
RpS3A	FBgn0264617	pipeline	pipeline	pipeline	RPS3A	1 mutant; viable
pan	FBgn0263851	II/III	lethal	viable	LEF1, TCF7L1	2 mutants; lethal
Ank	FBgn0085432	published	viable	viable	ANK1, ANK3	2 mutants; viable
anne	FBgn0011747	III	lethal	viable	ATP13A3, ATPA13A	4 mutants; lethal
CG32006	FBgn0264616	III	lethal	pipeline	FOXB1, FOXB2	pipeline
CG31997	FBgn0052006	II/III	viable	viable	no DIOPT homolog	2 mutants; viable; clones-P
CG33978	FBgn0051997	pipeline	viable	lethal	no DIOPT homolog	2 mutants; lethal
Arl4	FBgn0266725	II/III	viable	viable	ARL4A	2 mutants; viable
CG2316	FBgn0039889	II/III	viable	viable	ABCD1, ABCD2	3 mutants; viable
CG31998	FBgn0265634	II/III	viable	viable	pipeline	2 mutants; lethal
Crk	FBgn0051998	II	lethal	pipeline	CRK, CRKL	2 mutants; lethal
CG31999	FBgn0024811	II/III	viable	viable	FBN2, FBLN2	3 mutants; lethal
yellow-h	FBgn0051999	II/III	viable	viable	RGN	2 mutants; viable
CG1674	FBgn0267734	II/III	viable	viable	pipeline	3 mutants; viable
dpr7	FBgn0039897	II/III	lethal	viable	CD86	3 mutants; viable
RhoGAP102A	FBgn0053481	pipeline	viable	viable	ARHGAP6,	3 mutants; viable
Nfl	FBgn0259216	III	lethal	viable	NFIB	3 mutants; lethal
Syt7	FBgn0264793	published	viable	viable	SYT1, SYT2, SYT5	2 mutants; lethal
Rad23	FBgn0039900	published	viable	viable	RAD23A, RAD23B	3 mutants; viable
Zip102B	FBgn0026777	published	viable	pipeline	SLC39A8, SLC39A9	pipeline
CG32850/ Rnf11	FBgn0039902	II/III	lethal	lethal	RNF6, RNF11	2 mutants; viable
PMCA	FBgn0052850	II/III	lethal	viable	ATP2B2, ATP2B4	2 mutants; lethal
Hcf	FBgn0259214	III	lethal	viable	HCFC2, KLHDC4	2 mutants; lethal; clones-C
dati	FBgn0039904	III	lethal	lethal	ZNF362, ZNF384	1 mutant; lethal; clones-B
Igs	FBgn0264794	II/III	lethal	viable	pipeline	2 mutants; lethal; clones-E
CaMKI	FBgn0039907	II/III	viable	viable	CAMK1D, CAMK1G, CAMK2A, 2B, 2D, PNCK	3 mutants; viable
bip2 (Taf3)	FBgn0266619	II/III	lethal	pipeline	TAF3, TAF8	2 mutants; lethal
CG33941	FBgn0026262	II/III	viable	viable	no DIOPT homolog	2 mutants; viable
Asator DH.0			viable	viable	n/a	n/a
Asator DH.2	FBgn0262731	published	lethal	viable	TTBK1, TTBK2	2 mutants; lethal
zfh2	FBgn0039908	II/III	lethal	lethal	ZFHX2, ZFHX3	3 mutants; lethal; clones51A
Thd1/Tdg	FBgn0004607	pipeline	lethal	viable	TDG1	3 mutants; lethal
Pur-alpha	FBgn0264618	published	viable	viable	PURG, PURA	1 mutant; lethal; clones-B
ND-49	FBgn0022361	II/III	lethal	lethal	NDUFS1, NDUFS2	3 mutants; lethal; clones-G
Ephrin	FBgn0039909	pipeline	lethal	pipeline	EFNB2	3 mutants; viable; clones-A
CG1909	FBgn0040324	II/III	viable	viable	RAPSN	2 mutants; viable
onecut	FBgn0266726	III	lethal	viable	ONECUT1, ONECUT2	3 mutants; viable
Eph	FBgn0028996	III	viable	viable	EPHA1, EPHA2, EPHA8, EPHB1, EPHB2, EPHB6	2 mutants; viable
mav	FBgn0025936	II/III	viable	viable	TGFB1, TGFB2, TGFB3	3 mutants; viable

Gat	FBgn0262302	II/III	viable	viable	SLC6A5, SLC6A15, SLC6A19, SLC6A20	pipeline
Ekar	FBgn0039915	II / III	viable	viable	GIRK5, GRIK1	3 mutants; viable
gw	FBgn0264820	published	lethal	lethal	TNRC6A,TNRC6C	pipeline
Slip1	FBgn0051992	II/III	lethal	viable	pipeline	3 mutants; lethal; clones-A
CG11360	FBgn0024728	III	viable	viable	MEX3C, MEX3B	3 mutants; viable
myo	FBgn0039920	II/III	lethal	lethal	MSTN, GDF11	3 mutants; lethal; clones-D
ey	FBgn0026199	II/III	lethal	viable	PAX6, PAX7, PITX3, RAX, LHX6, GSC	3 mutants; lethal; clones-B
bt	FBgn0005558	pipeline	viable	viable	pipeline	2 mutants; lethal
MED26	FBgn0266727	pipeline	lethal	viable	MED26	3 mutants; lethal
Sox102F	FBgn0039923	III/X	viable	pipeline	SOX12, SOX17	2 mutants; viable
fd102C	FBgn0039938	published	viable	pipeline	FOXS1, FOXB1,	2 mutants; viable
Gyf	FBgn0266616	III/X	lethal	lethal	GIGYF	3 mutants; lethal
unc-13	FBgn0263344	published	viable	viable	UNC12A, UNC13B	2 mutants; lethal
eIF4G1	FBgn0263093	pipeline	lethal	pipeline	EIF4A1, EIF4G1,	3 mutants; lethal
mGluR DH.1		n/a	viable	viable	n/a	n/a
mGluR DH.2	FBgn0023213	II/III	viable	viable	GRM1, GRM4	2 mutants; viable; clones-A
4E-T	FBgn0019985	II/III	viable	viable	EIF4ENIF1	2 mutants; viable
fuss MI13731			viable	n/a	n/a	n/a
fuss MI03207	FBgn0264822	III	viable	viable	SKOR1, SKOR2	3 mutants; lethal
toy	FBgn0083990	II/III	lethal	viable	Same genes ey above	3 mutants; lethal
PlexA	FBgn0019650	III	lethal	lethal	PLXNA3, PLXNA4	2 mutants; lethal
CG11077/Mpv17	FBgn0264823	II/III	viable	pipeline	MPV17	3 mutants; viable
CG11076/Polr1G	FBgn0039930	II/III	lethal	pipeline	POLR1B	1 mutant; viable
ATPsynbeta	FBgn0039929	published	lethal	lethal	ATP5F1B	1 mutant; lethal
CaMKII	FBgn0010217	II	lethal	viable	Same genes CaMKI	3 mutants; lethal
Zyx	FBgn0264607	II	lethal	viable	LPP, ZYX	1 mutant; lethal
apolpp	FBgn0011642	II/III	lethal	lethal	APOE2, APOE3,	3 mutants; lethal; clones-B
Actbeta	FBgn0087002	II/III	lethal	viable	IHNA, INHBA, INHBB, INHBC, INHBE	2 mutants; lethal; clones-B
sv	FBgn0024913	II/III	lethal	lethal	Same genes ey above	2 mutants; viable; clones-F
Cals	FBgn0005561	II/III	viable	pipeline	CLSTN2	1 mutant; viable
Arf102F	FBgn0039928	II/III	lethal	pipeline	ARF5	1 mutant; lethal
CG11155/ukar			lethal	viable	n/a	n/a
CG11155/ukar	FBgn0013749	II/III	lethal	viable	GRIK5	3 mutants; viable
CG32017	FBgn0039927	II/III	viable	viable	no DIOPT homolog	3 mutants; lethal
Kif3C	FBgn0052017	II/III	viable	viable	KIF17	3 mutants; lethal; clones-A
pho	FBgn0039925	II/III	lethal	viable	YY1, YY2	1 mutant; lethal
CG33521	FBgn0002521	II/III	viable	viable	LIMA1, LIMD2, TRIP6	3 mutants; lethal
PIP4K	FBgn0250819	published	lethal	lethal	PIP4K2A, PIP5K1A	3 mutants; lethal
Mitf	FBgn0039924	published	lethal	pipeline	TFEB, TFE3, MITF, SREBF2	3 mutants; lethal
Dyrk3	FBgn0263112	II/III	viable	pipeline	DYRK1A, DYRK2	3 mutants; viable
Cadps	FBgn0027101	pipeline	lethal	viable	CADPS, CADPS2	2 mutants; lethal

Table S7

PCR primers for creating two new Tub.GAL80 insertions on FRT101F
**102B - Bloomington 606068 & Kyoto 119730**
102B pU6 gRNA forward<br>5' phosphorylated sense strand of the 102B gRNA to cloning into the pU6 plasmid by BbsI digestion & ligation<br>/5Phos/CTTCGAAATGCACATAATAGAGAT
102B pU6 gRNA reverse<br>5' phosphorylated anti-sense strand of the 102B gRNA to cloning into the pU6 plasmid by BbsI digestion & ligation<br>/5Phos/AAACATCTCTATTATGTGCATTTC
102B gRNA confirmation forward<br>Forward primer to amplify around the gRNA recognition sequence in the genome<br>TCCGCGAACCTCCATCAAAA
102B gRNA confirmation reverse<br>Reverse primer to amplify around the gRNA recognition sequence in the genome<br>AAGGGTAAGCGAAAGGTCGG
102B gRNA confirmation forward nested<br>Nested forward primer to amplify around & confirm the presence of the gRNA recognition sequence in the genome<br>TGAAAGGAAATTGGGCGGGT
102B gRNA confirmation reverse nested<br>Nested reverse primer to amplify around & confirm the presence of the gRNA recognition sequence in the genome<br>AGCAAGTAACCCACCTCTGC
102B HA2 outer forward<br>Forward primer to amplify the genomic region surrounding homology arm 2<br>TGTACGGCTTTCCCATGGAC
102B HA2 outer reverse<br>Reverse primer to amplify the genomic region surrounding homology arm 2<br>AACGATCGTCGCAGATGGAA
102B HA2 forward EcoRI<br>Forward primer to amplify homology arm 2, adds the EcoRI site to the 5' end<br>ATAATAGAATTCGATAAAGTACTTTTTTTTATTCATTGCTGTTTTTC
102B HA2 reverse BamHI<br>Reverse primer to amplify homology arm 2, adds BamHI site to the 3' end<br>TATTATGGATCCGGACCACTCCCTATACCCTTAC
tubGAL80 cassette forward Gibson<br>Forward primer to amplify the tubGAL80 cassette, adds overlap to the pUC19 plasmid for Gibson assembly<br>GTAAAACGACGGCCAGTGAATTCAAGCTTGCACAGGTCCTGTTC
102B tubGAL80 cassette reverse Gibson<br>Reverse primer to amplify the tubGAL80 cassette, adds overlap to the 102B homology arm 2 for Gibson assembly<br>GAAAAACAGCAATGAATAAAAAAAAGTACTTTATCGATCCAGACATGATAAGATACATTGATG
pUC19 reverse Gibson<br>Reverse primer to amplify the pUC19-HA2 plasmid for Gibson assembly with the tubGAL80 cassette<br>GAATTCACTGGCCGTCGTTTTAC
102B HA2 forward<br>Forward primer to amplify the pUC19-102B HA2 plasmid for Gibson assembly with the tubGAL80 cassette<br>GATAAAGTACTTTTTTTTATTCATTGCTGTTTTTC
tubGAL80 forward SapI<br>Forward primer to amplify the tubGAL80-HA2 cassette, adds SapI site to 5' end for cloning into pHD-DsRed<br>ATAATAGCTCTTCATATAAGCTTGCACAGGTCCTGTTC
102B HA2 reverse SapI<br>Reverse primer to amplify the tubGAL80-102B HA2 cassette, adds SapI site to 3' end for cloning into pHD-DsRed<br>ATAATAGCTCTTCAGACGGACCACTCCCTATACCCTTAC
102B CHK1 forward<br>Forward primer to amplify CHK 1 region of 102B<br>CCTGGAAACTGAAAGAAATACCCG
CHK1 reverse all gRNAs<br>Reverse primer to amplify CHK 1 region of all gRNAs<br>TTCTTGTAGTCGGGGATGTCGG

102B CHK1 forward nested<br>Nested forward primer to amplify & sequence CHK1 region of 102B homology arm1<br>CCTAGCTGGTGTTGCACTGA
CHK1 reverse all gRNAs nested<br>Nested reverse primer to amplify & sequence CHK 1 region of all gRNAs<br>TGGAGCCGTACTGGAACTG
102B CHK2 forward<br>Forward primer to amplify CHK 2 region of 102B<br>TCCGCGAACCTCCATCAAAA
CHK2 reverse all gRNAs<br>Reverse primer to amplify CHK 2 region of all gRNAs<br>TGAACTCCACCAGGTAGTG
102B CHK2 forward nested<br>Nested forward primer to amplify & sequence CHK 2 region of 102B<br>GCATAAGTCGACGACATCAC
CHK2 reverse all gRNAs nested<br>Reverse primer to amplify & sequence CHK 2 region of all gRNAs<br>CCGTCCTTCAGCTTCAGG
CHK3 forward all gRNAs<br>Forward primer to amplify CHK 3 region of all gRNAs<br>AAGAAGACTATGGGCTGGG
CHK3 reverse all gRNAs<br>Reverse primer to amplify CHK 3 region of all gRNAs<br>GTCACTCTAATCGCAGTCTG
CHK3 forward all gRNAs nested<br>Nested forward primer to amplify & sequence CHK 3 region of all gRNAs<br>GTGCTGAAGGGCGAGATCC
CHK3 reverse all gRNAs nested<br>Nested reverse primer to amplify & sequence CHK 3 region of all gRNAs<br>TACGTGTTTCCAACTTAACGCC
CHK4 forward all gRNAs<br>Forward primer to amplify CHK 4 region of all gRNAs<br>TACTGCCTTTCTGCGTTGG
CHK4 reverse all gRNAs<br>Reverse primer to amplify CHK 4 region of all gRNAs<br>GTGTCTTGATTGCCCATCC
CHK4 forward all gRNAs nested<br>Nested forward primer to amplify & sequence CHK 4 region of all gRNAs<br>AACATCGCCTTTTTCGTCC
CHK4 reverse all gRNAs nested<br>Nested reverse primer to amplify & sequence CHK 4 region of all gRNAs<br>CGTTGAGACCGACGAATC
CHK4 seq 1 all gRNAs<br>Additional sequencing primer #1 for CHK 4 region of all gRNAs<br>GCAAGTTTTAGAGAGACCAAGTGC
CHK4 seq 2 all gRNAs<br>Additional sequencing primer #2 for CHK 4 region of all gRNAs<br>TCGCAATGAGGAATGGCTC
CHK5 forward all gRNAs<br>Forward primer to amplify CHK 5 region of all gRNAs<br>TCAACGCAGCCAAAGGATGG
CHK5 reverse all gRNAs<br>Reverse primer to amplify CHK 5 region of all gRNAs<br>CACCACAGAAGTAAGGTTCC
CHK5 forward all gRNAs nested<br>Nested forward primer to amplify & sequence CHK 5 region of all gRNAs<br>ATCAAGACACATTACCCCGC

CHK5 reverse all gRNAs nested<br>Nested reverse primer to amplify & sequence CHK 5 region of all gRNAs<br>CAAGGTGGGAAAGCCTTCG
CHK6 forward all gRNAs<br>Forward primer to amplify CHK 6 region of all gRNAs<br>TGGCAATGTTCCAGTGTC
102B CHK6 reverse<br>Reverse primer to amplify CHK 6 region of 102B<br>AGCAAGTAACCCACCTCTGC
CHK6 forward all gRNAs nested<br>Nested forward primer to amplify & sequence CHK 6 region of all gRNAs<br>TGCAGTTTCAAAGGTGGC
102B CHK6 reverse nested<br>Nested reverse primer to amplify & sequence CHK 6 region of 102B<br>CCAACTACATTTGTGATGAACC
CHK6 seq 1 all gRNAs<br>Additional sequencing primer #1 for CHK 6 region of all gRNAs<br>CCTTACTTCTGTGGTGTGAC
CHK6 seq 2 all gRNAs<br>Additional sequencing primer #2 for CHK 6 region of all gRNAs<br>CAGCCATACCACATTTGTAGAG
CHK7 forward all gRNAs<br>Forward primer to amplify CHK 7 region of all gRNAs<br>CAGCCATACCACATTTGTAGAG
102B CHK7 reverse<br>Reverse primer to amplify CHK 7 region of 102B<br>AACGATCGTCGCAGATGGAA
102B CHK7 forward nested<br>Nested forward primer to amplify & sequence CHK 7 region of 102B<br>TGATGCGGACGATTTTAGTG
102B CHK7 reverse nested<br>Nested reverse primer to amplify & sequence CHK 7 region of 102B<br>TGCTGTAATGGGAGGATACG
**102C - 606069 Bloomington & 119731 Kyoto**
102C pU6 gRNA forward<br>5' phosphorylated sense strand of the 102C gRNA to cloning into the pU6 plasmid by BbsI digestion & ligation<br>/5Phos/CTTCGAATACGCTATTGTACTATC
102C pU6 gRNA reverse<br>5' phosphorylated anti-sense strand of the 102C gRNA to cloning into the pU6 plasmid by BbsI digestion & ligation<br>/5Phos/AAACGATAGTACAATAGCGTATTC
102C gRNA confirmation forward<br>Forward primer to amplify around the gRNA recognition sequence in the genome<br>GCGGAAGAAAGGTGGCAAAG
102C gRNA confirmation reverse<br>Reverse primer to amplify around the gRNA recognition sequence in the genome<br>CACGCCACCAAACGATTCAG
102C gRNA confirmation forward nested<br>Nested forward primer to amplify around & confirm the presence of the gRNA recognition sequence in the genome<br>TACCCTGTTTTCGACGCACA
102C gRNA confirmation reverse nested<br>Nested reverse primer to amplify around & confirm the presence of the gRNA recognition sequence in the genome<br>CTCGTTGCGTTCTGTGAAGC
102C HA2 outer forward<br>Forward primer to amplify the genomic region surrounding homology arm 2<br>GGCCAAATGTATGGGCGTTC
102C HA2 outer reverse<br>Reverse primer to amplify the genomic region surrounding homology arm 2<br>GGTCAAACAGTTGTCGACGC

102C HA2 forward EcoRI<br>Forward primer to amplify homology arm 2, adds the EcoRI site to the 5’ end<br>ATAATAGAATTCAGTACAATAGCGTATTCCTCG
102C HA2 reverse BamHI<br>Reverse primer to amplify homology arm 2, adds BamHI site to the 3’ end<br>TATTATGGATCCACGACGTGGTATTCACACTG
tubGAL80 cassette forward Gibson - As above
102C tubGAL80 cassette reverse Gibson<br>Reverse primer to amplify the tubGAL80 cassette, adds overlap with the 102C homology arm 2 for Gibson<br>GAAGTTCGAGGAATACGCTATTGTACTGATCCAGACATGATAAGATACATTGATG
102C HA2 forward<br>Forward primer to amplify the pUC19-102C HA2 plasmid for Gibson assembly with the tubGAL80 cassette<br>AGTACAATAGCGTATTCCTCGAACTTC
pUC19 reverse Gibson - As above
tubGAL80 forward SapI - As above
102C HA2 reverse SapI<br>Reverse primer to amplify the tubGAL80-102C HA2 cassette, adds SapI site to 3’ end for cloning into pHD-DsRed<br>ATAATAGCTCTTCAGACACGACGTGGTATTCACACTG
102C CHK1 forward<br>Forward primer to amplify CHK 1 region of 102C<br>TACCAATGCACGCATCTGCC
CHK1 reverse all gRNAs - As above
102C CHK1 forward nested<br>Nested forward primer to amplify & sequence CHK1 region of 102C homology arm1<br>TTCTGTTAGCAAACCTGAGTC
CHK1 reverse all gRNAs nested - As above
102C CHK2 forward<br>Forward primer to amplify CHK 2 region of 102C<br>TACCCTGTTTTCGACGCACA
CHK2 reverse all gRNAs - As above
102C CHK2 forward nested<br>Nested forward primer to amplify & sequence CHK 2 region of 102C<br>TGGCTTTCCTCTGCACCAC
CHK2 reverse all gRNAs nested - As above
CHK3 forward all gRNAs - As above
CHK3 reverse all gRNAs - As above
CHK3 forward all gRNAs nested - As above
CHK3 reverse all gRNAs nested - As above
CHK4 forward all gRNAs - As above
CHK4 reverse all gRNAs - As above
CHK4 forward all gRNAs nested - As above
CHK4 reverse all gRNAs nested - As above
CHK4 seq 1 all gRNAs - As above
CHK4 seq 2 all gRNAs - As above
CHK5 forward all gRNAs - As above
CHK5 reverse all gRNAs - As above
CHK5 forward all gRNAs nested - As above
CHK5 reverse all gRNAs nested - As above
CHK6 forward all gRNAs - As above
102C CHK6 reverse<br>Reverse primer to amplify CHK 6 region of 102C<br>CTCGTTGCGTTCTGTGAAGC
CHK6 forward all gRNAs nested - As above
102C CHK6 reverse nested<br>Nested reverse primer to amplify & sequence CHK 6 region of 102C<br>ACAGCCGAGCGGATTTGC
CHK6 seq 1 all gRNAs - As above

CHK6 seq 2 all gRNAs - As above
CHK7 forward all gRNAs - As above
102C CHK7 reverse<br>Reverse primer to amplify CHK 7 region of 102C<br>TATATCTCGGCACGCACGAAC
102C CHK7 forward nested<br>Nested forward primer to amplify & sequence CHK 7 region of 102C<br>CATGTCTGGATCAGTACAATAGCG
102C CHK7 reverse nested<br>Nested reverse primer to amplify & sequence CHK 7 region of 102C<br>GGTCAAACAGTTGTCGACGC
